# Discovering mutated driver genes through a robust and sparse co-regularized matrix factorization framework with prior information from mRNA expression patterns and interaction network

**DOI:** 10.1186/s12859-018-2218-y

**Published:** 2018-06-05

**Authors:** Jianing Xi, Minghui Wang, Ao Li

**Affiliations:** 1 School of Information Science and Technology, University of Science and Technology of China, Huangshan Road, Hefei, 230027 China; 2 Centers for Biomedical Engineering, University of Science and Technology of China, Huangshan Road, Hefei, 230027 China

**Keywords:** Driver gene, Network regularization, Matrix factorization, Cancer, Bioinformatics

## Abstract

**Background:**

Discovery of mutated driver genes is one of the primary objective for studying tumorigenesis. To discover some relatively low frequently mutated driver genes from somatic mutation data, many existing methods incorporate interaction network as prior information. However, the prior information of mRNA expression patterns are not exploited by these existing network-based methods, which is also proven to be highly informative of cancer progressions.

**Results:**

To incorporate prior information from both interaction network and mRNA expressions, we propose a robust and sparse co-regularized nonnegative matrix factorization to discover driver genes from mutation data. Furthermore, our framework also conducts Frobenius norm regularization to overcome overfitting issue. Sparsity-inducing penalty is employed to obtain sparse scores in gene representations, of which the top scored genes are selected as driver candidates. Evaluation experiments by known benchmarking genes indicate that the performance of our method benefits from the two type of prior information. Our method also outperforms the existing network-based methods, and detect some driver genes that are not predicted by the competing methods.

**Conclusions:**

In summary, our proposed method can improve the performance of driver gene discovery by effectively incorporating prior information from interaction network and mRNA expression patterns into a robust and sparse co-regularized matrix factorization framework.

**Electronic supplementary material:**

The online version of this article (10.1186/s12859-018-2218-y) contains supplementary material, which is available to authorized users.

## Background

To accelerate diagnostics and therapeutics of cancers, understand the causation of tumors is an urgent task [1]. Since cancer is a type of disease mainly caused by genomic aberrations, one of the primary objective for studying tumorigenesis is to discover mutated driver genes that can confer a selective survival advantage for tumor cells [1–3]. With the state-of-the-art technique next generation sequencing (NGS), enormous volume of DNA sequencing data of cancer cell samples have been increasingly accumulated [4–6]. Publicly available databases like The Cancer Genome Atlas (TCGA) [7] and the International Cancer Genome Consortium (ICGC) [8] have offered an unprecedented opportunity for the researches on cancer genomics. Nevertheless, despite the large amount of the somatic mutation data, there are many passenger mutations that are irrelevant to cancer phenotype, which greatly complicate the discovery of mutated driver genes [1, 9–11]. To discover mutated driver genes from sporadic passenger mutations, a straightforward way is to find highly mutated genes. Many previous methods use statistical test to compare the mutation rates of the tested genes with their background mutation rates, and select genes significantly highly mutated among the cancer samples [9, 12–15]. Moreover, MutSigCV [9] and CHASM [16] further predict cancer drivers based on multiple signals of positive selection and the functional impact.

Recently, a number of driver genes have been reported to be mutated with relatively low frequencies, and using only the mutated frequencies of genes may ignore some potential driver genes [3, 17, 18]. To detect driver genes with relatively low frequencies, many recently proposed methods are based on a prevalent assumption that mutated genes can perturb their interacted genes [17–22]. By incorporating interaction network of the genes as prior information, these methods detect mutated driver genes in the interacted network neighbors [23–26]. For example, HotNet and its revised version HotNet2 regard the mutated frequencies of genes as “heat” scores of the network nodes [17, 18]. By propagating the “heat” through the network, they can find not only highly mutated genes but also genes with relatively low mutated frequencies but important in network context. Another method called ReMIC identifies mutated driver genes through diffusion kernel of the network on mutational recurrences of the tested genes [19]. In addition to network propagation, MUFFINN investigates the mutational impact of genes by only their network neighbors, and considers either the highest mutated frequencies or the summation of all frequencies of the direct neighbors [21]. These network-based methods have pinpointed many novel mutated driver genes, which greatly expands the boundary of our understanding of driver events [3, 18, 21].

However, the existing methods aforementioned have not incorporated information from mRNA expression data, which are also widely available [27–32]. According to previous studies, mRNA expression data of tumor samples are capable of predicting clinical outcome of cancer patients [28–30] and survival-associated biomarkers [27, 31]. The altered mRNA expression profiles are also expected to reflect the molecular basis of the cancer patients, and the profiles are used as signatures for stratifying cancer patients with different survivals [33]. In addition to somatic mutations and interaction network, existing methods such as DriverNet [34] and DawnRank [35] also use mRNA expression information in driver gene detection task. Another method OncoIMPACT [36] further requires copy number alternations as its input variables. Instead of the direct usage of mRNA expressions aforementioned, the underlying similarities between cancer cell samples can also be computationally measured through mRNA expressions [37–40]. Notably, the expression based similarities are proven to be quite informative in several cancer related bioinformatics tasks such as drug-target interaction prediction [38], drug response prediction [40] and survival prediction [39]. Consequently, taking into consideration both expression pattern similarities between tumor samples and the interaction network information, the performance of discovering driver genes from mutation data could be potentially improved.

In this study, by incorporating somatic mutations, interaction network and mRNA expressions of genes, we introduce a novel and efficient method for predicting mutated driver genes. Motivated by a previous study [40], we model the similarities between tumor cells through their mRNA expression profiles into similarities between samples. The expression similarities of samples and gene interaction network are incorporated into an integrated framework based on graph co-regularized nonnegative matrix factorization (NMF) [41]. Furthermore, we also introduce Frobenius norm penalty to prevent overfitting issue [42], and sparsity-inducing penalty to obtain sparse representations of the mutated genes [43, 44]. When evaluated through two lists of known benchmarking driver genes [45, 46], our proposed method shows better detection results than the NMF methods with only gene interaction network, with only expression similarities of samples and with no prior information. We further compare our proposed method with existing network-based approaches for detecting driver genes, and find that our method yields the best performances among these competing approaches. Furthermore, the gene-set enrichment analysis [47] is also applied to determine whether members of a known driver gene set tend to occur toward the top of the genes detected by a method. By Fisher’s exact test, the gene-set enrichment results show that the genes detected by our methods are substantially more significant than those of the other competing approaches. Moreover, when we apply functional enrichment analysis on the detected genes, we find that most of the enriched pathways are related to cancer progressions. In addition, we also conduct literature survey and find some novel driver gene candidates from the results of our model.

## Methods

### Somatic mutation data and prior information

In this study, we use the somatic mutation data of three cancers from TCGA datasets [7], including glioblastoma multiforme (GBM) [48], colon and rectal cancer (COADREAD) [49] and breast cancer (BRCA) [50]. The reason why we select these three particular cancer types is that the numbers of known benchmarking genes of these three cancer types are relatively large for performance evaluation. To evaluate whether our model is generalizable for other cancer types as well, we further apply our model on the datasets of three other cancer types, kidney renal clear cell carcinoma (KIRC) [51], papillary thyroid carcinoma (THCA) [52] and prostate adenocarcinoma (PRAD) [53]. We download these datasets from a well-curated database cBioPortal [54]. The mutations of the cancer cell samples are then organized as a binary matrix (the entries of the matrix can be either one or zero), denoted as ***X***_*n*×*p*_ (when there are *n* samples and *p* genes for the input matrix) [19, 32, 55]. If the *j*-th gene of the *i*-th sample has a somatic mutation, then (*i*,*j*)-th entry of the matrix ***X***_*n*×*p*_ is set to one. The entry being zero represents no mutation found in the gene of the sample.

We also use mRNA expressions of genes as prior information. The data of mRNA expressions of the cancer samples aforementioned are also from TCGA datasets and downloaded from cBioPortal [54]. The gene expression data are normalized by median normalization by cBioPortal [54]. Since both somatic mutation data and mRNA expression data are used in this study, we use the cancer samples which have both mutation and expression data from TCGA datasets (82 samples for GBM, 207 samples for COADREAD, 503 samples for BRCA, 49 samples for KIRC, 390 samples for THCA and 333 samples for PRAD). By following previous work [40], we measure the similarities between cancer cell samples based on their gene expression patterns and form the sample similarity matrix *W*_*i*,*j*_= exp{−|1−*ρ*_*i*,*j*_|^2^/(2*σ*^2^)}, where *ρ*_*i*,*j*_ is the gene expression correlation between cancer samples. The parameter *σ* is bandwidth to control the extent of similarities fall off with the correlations, which is set to 1.0 in this study. When *ρ*_*i*,*j*_ is close to 0, *W*_*i*,*j*_ is also relatively small, giving only a weak contribution to the model. On the contrary, when the correlation *ρ*_*i*,*j*_ is close to 1, the similarity *W*_*i*,*j*_ is close to 1, too.

For the prior information of the gene interaction network, we use a highly curated interaction network iRefIndex [23]. We denote the adjacency matrix of the network as ***A***, of which the (*i*,*j*)-th entry being 1 represents the *i*-th gene and the *j*-th gene interact with each other. Since the interaction network is an undirected graph, the adjacency matrix ***A*** is a symmetric matrix. The degree matrix ***D***_***A***_ of the network is a diagonal matrix whose diagonal entries are the summation of the related rows (or columns) of matrix ***A***, i.e., $D_{i,i} = \sum _{j} A_{i,j}$. The Laplacian matrix of the network is defined as ***L***_***A***_=***D***_***A***_−***A***. For the sample similarity matrix ***W*** mentioned in the previous paragraph, we also calculate the Laplacian matrix ***L***_***W***_=***D***_***W***_−***W*** as same way as matrix ***L***_***A***_. Then, we use the symmetric normalization on the Laplacian matrix to obtain normalized Laplacian matrix $\boldsymbol {L}_{\boldsymbol {\hat {A}}} = \boldsymbol {D}_{\boldsymbol {A}}^{-1/2} \boldsymbol {L}_{\boldsymbol {A}} \boldsymbol {D}_{\boldsymbol {A}}^{-1/2} = \boldsymbol {I} - \boldsymbol {D}_{\boldsymbol {A}}^{-1/2} \boldsymbol {A} \boldsymbol {D}_{\boldsymbol {A}}^{-1/2}$, where the operation (·)^−1/2^ on a diagonal matrix is to replace the diagonal entries with the square root of them. We denote the matrix $\boldsymbol {\hat {A}} = \boldsymbol {D}_{\boldsymbol {A}}^{-1/2} \boldsymbol {A} \boldsymbol {D}_{\boldsymbol {A}}^{-1/2}$ as the normalized adjacency matrix of ***A***. In this situation, the normalized degree matrix $\boldsymbol {D}_{\boldsymbol {\hat {A}}}$ is reduced to the identity matrix. The ***L***_***W***_ matrix is not applied to the normalization process.

### Co-regularized NMF

The low-dimensional representations of different genes can be extracted by nonnegative matrix factorization (NMF) framework [41, 56, 57] from the somatic mutation matrix ***X***. In NMF, the sample gene matrix ***X*** can be decomposed into the matrix production of two low-rank nonnegative matrices ***U*** and ***V***. The reconstruction residual of matrix ***X*** is minimized in NMF, which is used to preserve the information of the input data:

1 $$\begin{array}{@{}rcl@{}} \min_{\boldsymbol{U} \in C_{u},\boldsymbol{V} \in C_{v}} \mathcal{L} (\boldsymbol{X},\boldsymbol{U V}^{\mathrm{T}}), \end{array} $$

where *C*_*u*_ and *C*_*v*_ are nonnegative constraint, which require the entries of the matrix to be nonnegative, and $\mathcal {L}$ is the loss function between the input data and the reconstructed data. $\boldsymbol {U} = \ [\!\boldsymbol {u}_{*,1}, \ldots, \boldsymbol {u}_{*,K}] =\ [\!\boldsymbol {u}_{1,*}, \ldots, \boldsymbol {u}_{n,*}]^{\mathrm {T}} \in \mathcal {R}^{n \times K}$ is the sample representation matrix, where *K* is the predefined dimension number of the latent representations. For ∀*k*∈{1,…,*K*}, the *k*-th vector ***u***_∗,*k*_ indicates the assignment weights of the cancer cell sample to the *k*-th latent dimension. The *i*-th ***u***_*i*,∗_ indicates the low-dimensional representations of the *i*-th cancer cell sample. $\boldsymbol {V} =\ [\!\boldsymbol {v}_{*,1}, \ldots, \boldsymbol {v}_{*,K}] =\ [\!\boldsymbol {v}_{1,*}, \ldots, \boldsymbol {v}_{p,*}]^{\mathrm {T}} \in \mathcal {R}^{p \times K}$ is the gene representation matrix, with the *k*-th vector ***v***_∗,*k*_ representing the weights of the tested genes in the *k*-th latent dimension. Each ***v***_*j*,∗_ denotes the representations of the tested genes in the latent dimension. NMF framework is also equivalent to maximizing the empirical likelihood of the input data [57].

For the biological interpretation of the low-dimensional representation of the samples, since the somatic mutation ***X***=[ ***x***_1,∗_,…,***x***_*i*,∗_,…,***x***_*n*,∗_]^T^ is composed of *n* vectors, we denote the *i*-th row vector ***x***_*i*,∗_ as the raw mutation profile of the *i*-th samples. The *k*-th vector ***v***_∗,*k*_ in matrix ***V*** can be regarded as the *k*-th latent mutation profile. Consequently, the loss function in Eq. (1) can be rewritten as $\mathcal {L} \left (\boldsymbol {x}_{i,*}, \sum _{k} u_{i,k} \boldsymbol {v}_{*,k}\right)$, i.e. minimizing the residuals between the raw mutation profile of the sample and the weighted sum reconstructed profile. Therefore, the raw mutation profile is approximated by the weighted sum of the latent mutation profiles, and the entries of low-dimensional representation of the samples are the proportions of the latent mutation profiles to combine the raw mutation profile.

Since the genes can be influenced by their interacted neighbors in the network, the preservation of the affinity in gene representations is an effective way for incorporating the prior information of the interaction network. Based on the local invariance assumption [41, 58, 59], if two genes interact with each other, then the distance of their representations ***v***_*i*,∗_ and ***v***_*j*,∗_ should also be small. The closeness between the low-dimensional representations of each pair of interacted genes can be measured by the graph regularization below [41, 60]

2 $$\begin{array}{@{}rcl@{}} R_{LV} (\boldsymbol{V}) = \sum\limits_{i=1}^{p} \sum\limits_{j=1}^{p} \ell \left(\boldsymbol{v}_{i,*},\boldsymbol{v}_{j,*}\right) \hat{A}_{i,j}. \end{array} $$

Due to the similarity of expression patterns between the cancer cell samples, we also incorporate the sample-wise similarities into the low-dimensional representations of samples. Similar to the representations of genes, if two cancer cell samples are similar in their expression patterns, then their low-dimensional representations ***u***_*i*,∗_ and ***u***_*j*,∗_ should also be close. To achieve the closeness between the representations. we introduce the following graph regularization

3 $$\begin{array}{@{}rcl@{}} R_{LU} (\boldsymbol{U}) = \sum\limits_{i=1}^{n} \sum\limits_{j=1}^{n} \ell (\boldsymbol{u}_{i,*},\boldsymbol{u}_{j,*}) W_{i,j}. \end{array} $$

The two terms of graph regularization in both Eqs. (2) and (3) are referred as graph co-regularization, due to the fact that they simultaneously preserve the affinity on samples and genes. They are used to incorporate prior information of both cancer sample similarity and gene interaction network into the latent factors.

When we combine together the NMF low-dimensional representation and the closeness between the samples/genes, we yield the objective function of co-regularized NMF (CRNMF) [41] as shown below

4 $$\begin{array}{@{}rcl@{}} \min_{\boldsymbol{U} \in C_{u}, \boldsymbol{V} \in C_{v}} \mathcal{L} \left(\boldsymbol{X}, \boldsymbol{U V}^{\mathrm{T}}\right) + \lambda_{LU} R_{LU} (\boldsymbol{U}) + \lambda_{LV} R_{LV} (\boldsymbol{V}) \end{array} $$

where *λ*_*LU*_ and *λ*_*LV*_ are the graph regularization parameters for samples and genes respectively. There are three reasons to integrate the two learning objectives into one optimization framework seamlessly. First, the common latent low-dimensional representations are extracted from somatic mutation data through NMF [41]. Second, the prior information of gene interaction network and tumor sample similarity are incorporated in the representations through graph co-regularization. Third, graph co-regularization and matrix factorization can be simultaneously performed to learn the representations preserving both the information of the original data and geometric structure of affinity, where the learned representations can approximately recover the original data through matrix multiplication, and the distance between the representations of two similar samples or two interacted genes are also close to each other.

### Robust and sparse CRNMF

**Fig. 1:**
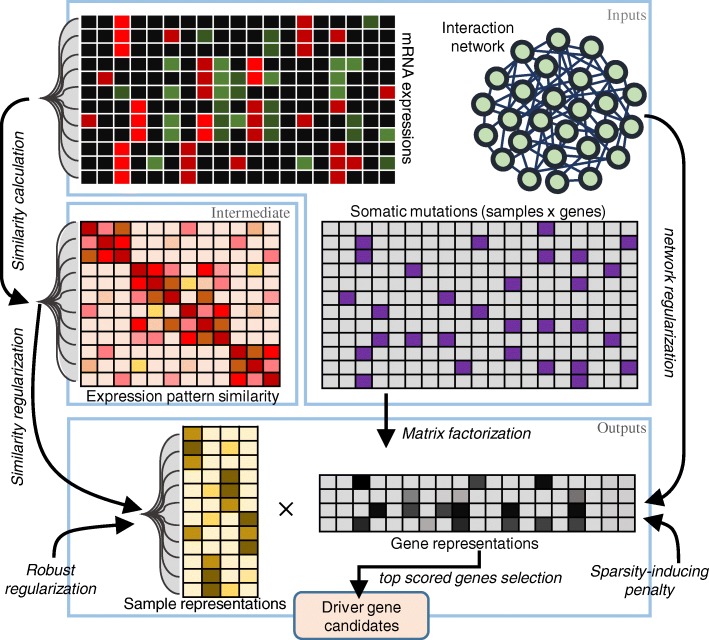
Schematic diagram of the proposed method. For discovering driver genes from somatic mutation data, we propose a robust and sparse co-regularized NMF framework by incorporating prior information of both mRNA expression patterns and interaction network. The input data contain three parts: 1) the binary somatic mutation matrix of cancer samples and genes, 2) the mRNA expression matrix of cancer samples and genes, and 3) the interaction network of genes. The mRNA expression patterns are used to calculate the sample similarities between tumor samples, which is used as the intermediate variable. We then use NMF co-regularized by the sample similarity and gene interaction network to incorporate their prior information. Robust regularization are employed to prevent overfitting issue for the representation of samples, and sparsity-inducing penalty is also used to generate sparse representation of genes. The tested genes are scored through the maximal values in their low-dimensional representations, and the top scored genes are selected as driver candidates

In this subsection, we introduce our proposed method robust and sparse CRNMF, of which the schematic diagram is illustrated in Fig. 1. Different from CRNMF, our method also considers two important aspects on the low-dimensional representations of both samples and genes. One aspect is the overfitting issue [42]. To adequately exploit the input data and achieve a more generalization model, we need to prevent some extreme values in the samples representations, which may cause that the reconstruction of input data are contributed by only a small number of samples rather than all samples [42]. Another aspect is that most genes are not related to cancer progressions and only a few genes are driver genes [1, 9, 10]. Consequently, the values of gene representations are required to be sparse. In other word, for each latent dimension, the representation value of only a small proportion of the genes are expected to be larger than zero [43, 44].

We introduce two regularization terms to quantitatively measure the two aspects. First, the overfitting problem of sample representations can be measured by whether they are some extreme values, denoted as *R*_*O*_(***U***)=*f*(***U***). Here *f*(·) represent a nonlinear transformation, which can amplify larger input values and attenuate small input values [42]. This property makes the regularization term intolerant for very large values, and minimizing this term can prevent the sample representations from extreme values. Second, the sparseness of the values in gene representation can be obtained by sparsity-inducing penalty term $R_{S}(\boldsymbol {V}) = \sum _{k=1}^{K} g(\boldsymbol {v}_{*,k})$ [43, 44]. When the function *g*(·) is sensitive to small values, it can penalize the small values in the gene representation and lead to sparseness [61]. When *g*(·) is a convex function, the optimization procedure can be facilitated by the convexity property [43, 44, 61]. We rewrite the objective function of robust and sparse CRNMF as below, where the parameters *λ*_*RV*_ and *λ*_*RV*_ are the tuning parameters for robust regularization on matrix ***U*** and sparse regularization ***V*** respectively

5 $$\begin{array}{@{}rcl@{}} \begin{aligned} \min_{\boldsymbol{U} \in C_{u}, \boldsymbol{V} \in C_{v}} & \mathcal{L} \left(\boldsymbol{X}, \boldsymbol{U V}^{\mathrm{T}}\right) + \lambda_{LU} R_{LU} (\boldsymbol{U}) + \lambda_{RU} R_{O}\left(f(\boldsymbol{U})\right) \\ & + \lambda_{LV} R_{LV} (\boldsymbol{V}) + \lambda_{RV} R_{S} (\boldsymbol{V}). \end{aligned} \end{array} $$

The aforementioned framework is a general formulation, where various loss functions $\mathcal {L}$, *ℓ*, *f* and *g* can be chosen from different options. Their options used in this study are as follows: Loss function $\mathcal {L}$ used in matrix factorization is the summation of squares loss, $\mathcal {L} (\boldsymbol {X},\boldsymbol {\hat {X}}) = \left \| \boldsymbol {X} - \boldsymbol {\hat {X}} \right \|_{F}^{2}$. Loss function *ℓ* is the Euclidian distance, i.e., $\ell (\boldsymbol {x}, \boldsymbol {\hat {x}}) = \left \| \boldsymbol {x} - \boldsymbol {\hat {x}} \right \|_{2}^{2}$. In this case, the graph regularization terms can be reformed as

6 $$\begin{array}{@{}rcl@{}} \begin{aligned} R_{LU}(\boldsymbol{U}) & = \sum\limits_{i=1}^{n} \sum\limits_{j=1}^{n} \left(\boldsymbol{u}_{i,*}^{\mathrm{T}} \boldsymbol{u}_{j,*}\right) (\boldsymbol{L}_{\boldsymbol{W}})_{i,j} = \text{Tr} \left\{\boldsymbol{U}^{\mathrm{T}} \boldsymbol{L}_{\boldsymbol{W}} \boldsymbol{U} \right\}\\ R_{LV}(\boldsymbol{V}) & = \sum\limits_{i=1}^{p} \sum\limits_{j=1}^{p} \left(\boldsymbol{v}_{i,*}^{\mathrm{T}} \boldsymbol{v}_{j,*}\right) \left(\boldsymbol{L}_{\boldsymbol{\hat{A}}}\right)_{i,j} = \text{Tr}\left \{ \boldsymbol{V}^{\mathrm{T}} \boldsymbol{L}_{\boldsymbol{\hat{A}}} \boldsymbol{V} \right\} \end{aligned} \end{array} $$

For the robust regularization, we choose squared Frobenius norm [42] as the nonlinear transformation. The squared Frobenius norm is equivalent to the summation of the square of the entries, i.e., $\left \| \boldsymbol {U} \right \|_{F}^{2} = \sum _{i} \sum _{j} \left (U_{i,j}\right)^{2}$, which satisfies the property of intolerance for very large values. For the sparsity-inducing penalty term, we use the squared L1-norm as the function for the input vector $g(\boldsymbol {v}_{*,k}) = \left \| \boldsymbol {v}_{*,k} \right \|_{1}^{2} = \left (\sum _{j} |v_{j,k}|\right)^{2}$, since the L1-norm is convex function and is also one of the most widely used sparsity-inducing loss in previous studies [43, 44]. Using the settings above, the framework in Eq. (5) is formed as

7 $$\begin{array}{@{}rcl@{}} \begin{aligned} \min_{\boldsymbol{U} \leq 0, \boldsymbol{V} \leq 0} & \left\| \boldsymbol{X} - \boldsymbol{\hat{X}} \right\|_{F}^{2} + \lambda_{LU} \text{Tr} \{\boldsymbol{U}^{\mathrm{T}} \boldsymbol{L}_{\boldsymbol{W}} \boldsymbol{U} \} + \lambda_{RU} \left\| \boldsymbol{U} \right\|_{F}^{2}\\ & + \lambda_{LV} \text{Tr} \{ \boldsymbol{V}^{\mathrm{T}} \boldsymbol{L}_{\boldsymbol{\hat{A}}} \boldsymbol{V} \} + \lambda_{RV} \sum\limits_{k=1}^{K} \left\| \boldsymbol{v}_{*,k} \right\|_{1}^{2}. \end{aligned} \end{array} $$

The objective function in Eq. (7) can be solved by an alternating optimization procedure, as shown below,

8 $$\begin{array}{@{}rcl@{}} U_{i,j} \leftarrow U_{i,j} \frac{(\boldsymbol{X} \boldsymbol{V} + \lambda_{LU} \boldsymbol{W} \boldsymbol{U})_{i,j}}{\left(\boldsymbol{U V}^{\mathrm{T}} \boldsymbol{V} + \lambda_{LU} \boldsymbol{D}_{\boldsymbol{W}} \boldsymbol{U} + \lambda_{RU} \boldsymbol{U}\right)_{i,j}} \end{array} $$

9 $$\begin{array}{@{}rcl@{}} V_{i,j} \leftarrow V_{i,j} \frac{\left(\boldsymbol{X}^{\mathrm{T}} \boldsymbol{U} + \lambda_{LV} \boldsymbol{\hat{A}} \boldsymbol{V}\right)_{i,j}}{\left(\boldsymbol{V U}^{\mathrm{T}} \boldsymbol{U} + \lambda_{LV} \boldsymbol{D}_{\boldsymbol{\hat{A}}} \boldsymbol{V} + \lambda_{RV} \boldsymbol{E}_{p \times p} \boldsymbol{V}\right)_{i,j}} \end{array} $$

where ***E***_*p*×*p*_ is a *p* by *p* matrix with all entries being 1. In this study, the dimension number of the latent representations *K* is set to 4 and the tuning parameters *λ*_*LU*_, *λ*_*RU*_, *λ*_*LV*_ and *λ*_*RV*_ are set to 1.0 as suggested by a previous study [32], which also uses NMF framework and graph regularization on somatic mutation data of cancers. For the source code of the method in GitHub, we have also offered the options for users to set the parameters separately for their own applications. Furthermore, we evaluate the performance of the model when the number of dimensions increases, as shown in Additional file 1: Figures S1. The evaluation show that the performance of our model varies slightly among these numbers of dimensions, indicating that our model are not sensitive to the number of dimensions.

Through the usage of updating rules of ***U*** and ***V*** in Eqs. (8) and (9) sequentially, the objective function in Eq. (7) can be decreased until convergence. Finally, to discover driver genes, we use the maximum values in the low-dimensional representation of each tested gene as its mutation score, and prioritize the tested genes by their mutation scores. Rather than using the average value across the dimensions as the score of each gene, we use the maximum coefficient across the dimensions, which can reflect the mutation score of each gene in a subset of samples and is more effective for heterogeneous cancers.

## Results

### Evaluation metrics

In this study, we use two lists of well-curated benchmarking driver genes to evaluate the performance of our approach in the discovery of driver genes. The first benchmarking gene list used for evaluation is the 537 known driver genes curated by Cancer Gene Census (CGC) which are experimentally supported [45]. The cancer types related to these genes are also provided by CGC database. The second benchmarking gene list is from another independent database of cancer drivers called Integrative Onco Genomics (IntOGen) [46]. By regarding the benchmarking genes from the two independent lists as ground truths, we can comprehensively evaluate the performance of driver gene discovery.

To quantitatively assess the performance, we introduce evaluation metrics precision = TP/TP+FP, recall = TP/TP+FN. Due to the fact that known driver genes are much less than the other genes in the discovery of driver genes, in the evaluation, precision is more sensitive to false positive than recall. By draw precisions against recalls over different cutoff ranks, we can obtain precision recall curves of the discovery results, where a higher curve denotes a better performance [62, 63]. For a precision recall curve, the area under the curve (AUC) is also larger when the discovery performance is better, which can also be used for assessment. Since only the top scored candidates might be validated by experimental follow-up [21], the top 200 genes are selected as the driver gene candidates, as suggested in a previous study [22]. To assess whether the numbers of benchmarking genes in top scored candidates are significantly different from random selections, we also employ the Fisher’s exact test on the top scored genes of the discovered results.

### Comparison analysis of prior information

To assess the contribution of prior information used in our proposed approach, we firstly compare our method to the NMF methods with only one of the two kinds of information and with no prior information. When we set the tuning parameter *λ*_*LU*_ and *λ*_*RU*_ in Eq. (7) to zero, we can obtain NMF with only network information. Similarity, we can yield NMF with only information from expression pattern similarity by setting the tuning parameter *λ*_*LV*_ and *λ*_*RV*_ in Eq. (7) to zero. In the situation that both the four tuning parameters are set to zero, the framework in Eq. (7) is reduced to original NMF with no prior information. In brief, we denote our proposed method, NMF with only network information, NMF with only expression pattern information and NMF with no prior information as “Proposed”, “Only network”, “Only expression” and “No prior” respectively in the following paragraphs.

**Fig. 2:**
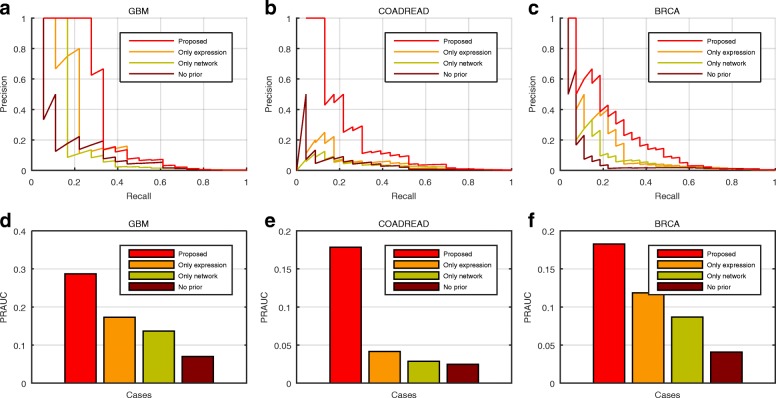
Performance comparison of NMF with different kinds of prior information, evaluated by CGC list [45]. The precision recall curves of the results of our proposed method (“Proposed”: red), NMF with information of mRNA expression pattern similarity (“Only expression”: orange), NMF with only network information (“Only network”: yellow), and NMF with no prior information (“No prior”: dark red), for datatsets of (**a**) GBM, (**b**) COADREAD and (**c**) BRCA. The AUCs of precision recall curves of “Proposed”, “Only expression”, “Only network” and “No prior”, displayed as bar plot, for datatsets of (**d**) GBM, (**e**) COADREAD and (**f**) BRCA

Through the precision recall curves of the NMF based methods with different prior information in Fig. 2a–c, we can observe that our proposed model outperforms the other NMF methods with at least one of the two types of information removed. When applied on GBM dataset and evaluated by CGC gene list, our proposed method achieve a AUC of 28.7%, compared with 13.7% of “Only network”, 17.3% of “Only expression” and 7.0% of “No prior” (Fig. 2d). The AUCs of our method on COADREAD and BRCA are 17.8 and 18.3% (Fig. 2e–f), which are also higher than those of the other three methods in the same situations. Furthermore, we display the precision recall curves based on IntOGen list (Additional file 1: Figure S2(a)-(c)), we can obtain same conclusion that the proposed method yields higher performance than those of “Only network”, “Only expression” and “No prior” on GBM, COADREAD and BRCA data. For example, the AUCs of our method on GBM, COADREAD and BRCA are 11.4%, 9.8% and 13.5% respectively (Additional file 1: Figure S2(d)-(f)), and their values are also larger than those of “Only network”, “Only expression” and “No prior”. To clearly evaluate whether the improvement is from the prior knowledge, we further demonstrate the results of our methods when the parameters for sparseness (or robustness) are fixed and the parameters for prior knowledge varies, i.e., the case where *λ*_*RU*_ is fixed and *λ*_*LU*_ varies (Additional file 1: Figures S3) and the case where *λ*_*RV*_ is fixed and *λ*_*LV*_ varies (Additional file 1: Figures S4). We can observe that the performance of our methods also increase when the tuning parameters for prior knowledge increase in most situations, indicating that the improvement is from the prior knowledge.

### Comparison with existing methods

In this subsection, we compare our method with five previous published methods, DriverNet [34], DawnRank [35], HotNet2 [18], ReMIC [19] and MUFFINN [21]. In the comparison, DawnRank, DriverNet and HotNet2 are set with their default parameters [18, 34, 35]. For ReMIC, we follow the previous work and set the diffusion strength *β* to three values 0.01, 0.02 and 0.03 [19]. Both of the two different versions of MUFFINN are used in this study, known as MUFFINN(DNmax) and MUFFINN(DNsum) [21]. For all the five existing network-based methods, we also use iRefIndex as prior information from network as is used in our method [23].

**Fig. 3:**
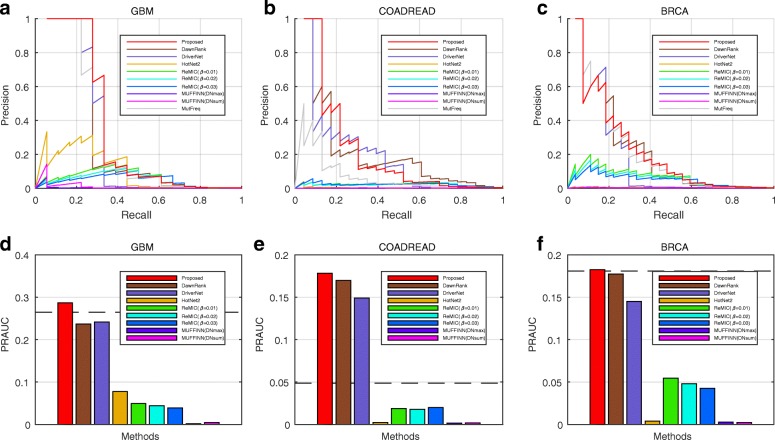
Performance comparison of our proposed method and existing network-based methods, evaluated by CGC list [45]. The precision recall curves of the results of our proposed method (red), DawnRank (brown), DriverNet (medium purple), HotNet2 (orange), ReMIC(*β*=0.01) (green), ReMIC(*β*=0.02) (cyan), ReMIC(*β*=0.03) (blue), MUFFINN(DNmax) (violet), MUFFINN(DNsum) (magenta) and baseline by mutation frequency (gray), for datatsets of (**a**) GBM, (**b**) COADREAD and (**c**) BRCA. The AUCs of precision recall curves of the competing methods, displayed as bar plot, for datatsets of (**d**) GBM, (**e**) COADREAD and (**f**) BRCA. The black dash lines in (**d**)–(**f**) represent the AUC values of baseline by mutation frequency

The precision recall curves of the competing methods are illustrated in Fig. 3a–c for CGC evaluation and Additional file 1: Figure S5(a)-(c) for IntOGen evaluation. Since most of the validated benchmarking genes are curated based on high mutation frequencies [1, 45, 46], the performance calculated by mutation frequencies can be regarded as baseline performance, and our model achieves higher performance against the baseline performance. Compared with these existing network-based methods, the discovery results of our proposed method are largely elevated, for the evaluation of CGC benchmarking lists. Taking GBM as an example, the AUC of DawnRank, DriverNet, HotNet2, ReMIC (*β*= 0.01), ReMIC (*β*= 0.02), ReMIC (*β*= 0.03), MUFFINN(DNmax) and MUFFINN(DNsum) are 23.7%, 24.1%, 7.8%, 5.0%, 4.4%, 3.9%, 0.2% and 0.5% respectively, when evaluated by CGC list (Fig. 3d). In comparison, our proposed method achieves a AUC of 28.7% evaluated by CGC, which is larger than the values of the results of the existing methods. For IntOGen evaluation, the AUCs for GBM achieved by DawnRank, DriverNet, HotNet2, ReMIC (*β*= 0.01), ReMIC (*β*= 0.02), ReMIC (*β*= 0.03), MUFFINN(DNmax) and MUFFINN(DNsum) are 10.4%, 8.3%, 3.8%, 3.2%, 3.2%, 2.9%, 0.7% and 0.8% respectively, while the AUC of our method is 11.4% (Additional file 1: Figure S5(d)). For COADREAD and BRCA data, the AUCs of our method are also comparable or larger than the AUCs of the competing approaches, when evaluated by both CGC (Fig. 3e–f) and IntOGen lists (Additional file 1: Figure S5(e)-(f)). In addition, we also demonstrate the results of the comparison methods on the three other cancer types KIRC, THCA and PRAD. The results show that our model also performs comparable or better than the comparison methods when applied on the datasets of the three other cancer types (Additional file 1: Figures S6-S7).

Furthermore, we also investigate the top scored driver candidates discovered by the competing methods. By applying the gene-set enrichment analysis [47], we test whether the top scored genes of our methods are significantly different from random selections of the genes in the two benchmarking lists, when the threshold are 50, 100, 150 and 200 (Table 1). For example, for the top 200 genes, when we employ the significant test on the results for COADREAD data, the enrichment *p*-values of HotNet2, ReMIC(*β*= 0.01), ReMIC(*β*= 0.02), ReMIC(*β*= 0.03) on COADREAD data are 5.46e-02, 3.06e-05, 4.97e-04 and 4.97e-04 respectively. In comparison, the *p*-values of our method is 3.35e-16. When we investigate the *p*-values of the top scored genes of these methods for IntOGen, the enrichment *p*-values of our method for top 200 genes is 1.30e-18, which is also smaller than the *p*-values of the other competing methods. For GBM and BRCA data, we can observe similar phenomenon that the discovery results of our proposed method are significantly enriched for benchmarking gene lists of both CGC and IntOGen (Additional file 1: Table S1-S2).

**Table 1:** Fisher’s exact test on the top scored candidates of COADREAD results for CGC and IntOGen benchmarking genes

	CGC				IntOGen			
Top	50	100	150	200	50	100	150	200
Proposed	3.05e-12	9.59e-16	9.86e-18	3.35e-16	1.66e-15	2.48e-17	1.88e-18	1.30e-18
HotNet2	9.07e-02	1.74e-01	2.49e-01	5.46e-02	5.51e-02	1.76e-01	3.15e-01	1.94e-01
ReMIC(*β* = 0.01)	9.07e-02	1.52e-02	2.74e-03	3.06e-05	8.78e-08	4.77e-13	3.45e-13	7.59e-15
ReMIC(*β* = 0.02)	9.07e-02	1.52e-02	2.74e-03	4.97e-04	8.78e-08	4.77e-13	3.45e-13	7.59e-15
ReMIC(*β* = 0.03)	3.99e-03	8.54e-04	1.66e-04	4.97e-04	1.96e-06	2.11e-10	3.45e-13	1.72e-12
MUFFINN(DNmax)	1.00e-00	1.00e-00	1.00e-00	1.00e-00	1.00e-00	1.00e-00	1.00e-00	1.00e-00
MUFFINN(DNmax)	1.00e-00	1.00e-00	1.00e-00	1.00e-00	1.00e-00	1.00e-00	6.32e-01	4.09e-01

The *p*-values are for the results our proposed method, HotNet2, ReMIC(*β*=0.01), ReMIC(*β*=0.02), ReMIC(*β*=0.03), MUFFINN(DNmax) and MUFFINN(DNsum)

**Fig. 4:**
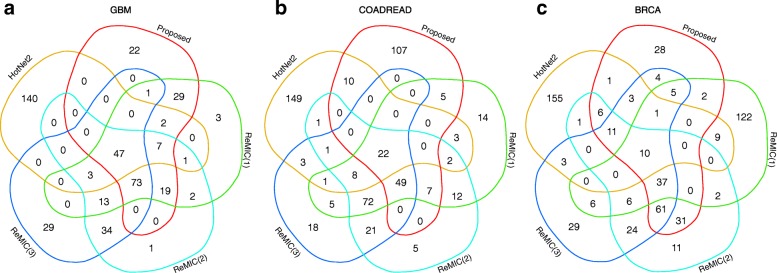
Venn diagrams of the top scored genes of some of the competing methods. The diagram illustrate the relations among the top 200 candidates in the results of our proposed method (red), HotNet2 (orange), ReMIC(*β*=0.01) (green), ReMIC(*β*=0.02) (cyan), ReMIC(*β*=0.03) (blue) on (**a**) GBM, (**b**) COADREAD and (**c**) GBM datasets

We also demonstrate Venn diagram (Fig. 4) among the top 200 genes of some of the competing methods. For all the three cancer datasets, we can observe a relatively high concordance between the our results and the results of the other network-based methods. Among the top 200 genes of these methods, there are 89.0% (GBM), 46.5% (COADREAD) and 86.0% (BRCA) genes detected by our proposed methods which are also included in the top scored genes discovered by at least one of the other network-based methods. For example, the five results on GBM dataset share 47 common genes, including *TP53*, *PTEN*, *BRCA2* that are curated by both CGC and IntOGen (Supplementary Table). These five results also share CGC gene *APC* for COADREAD data and IntOGen gene *ANK3* for BRCA data (Supplementary Table). Meanwhile, there are also some driver are found by only our proposed method. For example, known CGC genes *PIK3CA*, *TP53* and IntOGen genes *HDAC9*, *KALRN*, *LRP6*, *MAP3K4* and *TGFBR2* are found by only our method for COADREAD (Supplementary Table). For BRCA, CGC gene *PTEN* and IntOGen gene *RB1* and *SF3B1* are unique to the result of our proposed method (Supplementary Table). The full lists of the top 200 genes for GBM, COADREAD and BRCA discovered by our method are provided in Additional file 1: Table S3-S5 respectively.

### Functional enrichment analysis

In addition to the evaluation of benchmarking genes, functional enrichment analysis is another way to assess the association between the top scored genes and cancer progressions. Here we apply functional enrichment analysis for the Kyoto Encyclopedia of Genes and Genomes (KEGG) pathways [64] on the top 200 driver candidates to find whether their shared biological functions are also correlated with cancer. For GBM, the driver gene candidates are highly enriched for cancer related pathways (Table 2), such as Pathway in cancer (*p* = 1.44e-24), Glioma (*p* = 5.09e-24), Melanoma (*p* = 1.41e-09), p53 signaling pathway (*p* = 8.11e-09) and mTOR signaling pathway (*p* = 2.29e-06). For COADREAD, the top scored genes are highly associated with pathways like Focal adhesion (*p* = 2.15e-09), Pathways in cancer (*p* = 2.45e-09), Colorectal cancer (*p* = 7.18e-09), Pancreatic cancer (*p* = 1.61e-06) Prostate cancer (*p* = 2.66e-06) and Renal cell carcinoma (*p* = 9.05e-04) (Additional file 1: Table S6). For BRCA result, the top 200 genes are significantly enriched for Calcium signaling pathway (*p* = 3.11e-07), Focal adhesion (*p* = 3.46e-07), ErbB signaling pathway (*p* = 1.53e-05), Endometrial cancer (*p* = 2.51e-05), MAPK signaling pathway (*p* = 3.79e-04) and Apoptosis (*p* = 6.15e-04) (Additional file 1: Table S7).

**Table 2:** Functional enrichment analysis results for KEGG pathways [64] of the top 200 genes of the proposed method on GBM dataset

Pathway	Count	%	*p*-value	Pathway	Count	%	*p*-value
Pathways in cancer	48	24.12	1.44e-24	Leukocyte transendothelial migration	11	5.53	1.44e-04
Focal adhesion	28	14.07	5.09e-15	Apoptosis	8	4.02	2.55e-04
Prostate cancer	20	10.05	6.97e-15	Renal cell carcinoma	8	4.02	3.43e-04
Glioma	15	7.54	3.13e-11	Gap junction	9	4.52	4.20e-04
Pancreatic cancer	15	7.54	3.13e-11	Melanogenesis	9	4.52	9.88e-04
Colorectal cancer	14	7.04	2.35e-10	Small cell lung cancer	8	4.02	1.73e-03
Melanoma	14	7.04	1.41e-09	Wnt signaling pathway	10	5.03	2.06e-03
Endometrial cancer	12	6.03	5.50e-09	Hedgehog signaling pathway	5	2.51	2.08e-03
p53 signaling pathway	13	6.53	8.11e-09	Natural killer cell mediated cytotoxicity	9	4.52	3.50e-03
Non-small cell lung cancer	12	6.03	1.26e-08	Chemokine signaling pathway	11	5.53	4.82e-03
Chronic myeloid leukemia	13	6.53	1.90e-08	Endocytosis	13	6.53	6.63e-03
Neurotrophin signaling pathway	15	7.54	1.31e-07	Fc gamma R-mediated phagocytosis	7	3.52	7.40e-03
Regulation of actin cytoskeleton	18	9.05	1.26e-06	Jak-STAT signaling pathway	9	4.52	9.78e-03
ErbB signaling pathway	12	6.03	1.38e-06	Mismatch repair	4	2.01	1.11e-02
Acute myeloid leukemia	10	5.03	1.69e-06	Calcium signaling pathway	10	5.03	1.12e-02
mTOR signaling pathway	10	5.03	2.29e-06	B cell receptor signaling pathway	6	3.02	1.34e-02
Cell cycle	13	6.53	7.91e-06	Adipocytokine signaling pathway	6	3.02	1.42e-02
Fc epsilon RI signaling pathway	10	5.03	8.92e-06	T cell receptor signaling pathway	7	3.52	1.90e-02
Adherens junction	10	5.03	1.28e-05	Cytokine-cytokine receptor interaction	11	5.53	1.97e-02
Bladder cancer	8	4.02	1.67e-05	Thyroid cancer	4	2.01	2.10e-02
Insulin signaling pathway	13	6.53	2.36e-05	Tight junction	8	4.02	2.24e-02
VEGF signaling pathway	9	4.52	3.07e-05	Phosphatidylinositol signaling system	6	3.02	5.07e-02
MAPK signaling pathway	17	8.54	6.19e-05	Toll-like receptor signaling pathway	6	3.02	6.67e-02
GnRH signaling pathway	10	5.03	9.50e-05	Notch signaling pathway	4	2.01	7.52e-02
Basal cell carcinoma	8	4.02	1.19e-04	TGF-beta signaling pathway	5	2.51	9.39e-02

The pathways are sorted by their enrichment *p*-values

### Literature survey

To investigate whether there are some novel insights that can be learned from the model for each cancer type, we further conduct a literature survey on the genes detected by our model that are not annotated in the benchmarking lists. For GBM results, *ERBB2* is detected as one of the top ranked genes. Although *ERBB2* is recognized as driver gene for several cancer types, but it is not curated as GBM driver gene in the two benchmarking lists [45, 46]. However, a recent study shows that *ERBB2* mutations are associated with GBM formation and progression [65]. *MSH6* is another gene detected in GBM results. Recent studies have reported that *MSH6* mutations are considered to play an important role in the recurrence of glioma, acquired resistance to alkylating agents and genome instability [66, 67]. Moreover, *TERT* is also found as a driver gene candidate by our model in GBM results, although *TERT* is not included in the 537 CGC genes either. Recent research has shown that *TERT* mutations are observed in the most aggressive human glioma (grade IV astrocytoma) and the least aggressive diffuse human glioma (grade II oligodendroglioma) at the same time [68].

For COADREAD results, *SYNE1* is the top 5 gene detected by our model. Mutations in *SYNE1* are reported to be associated with colorectal cancers in previous studies [69]. Meanwhile, another recent study has observed high prevalence of non-silent mutations in *SYNE1* among 160 colorectal cancer patients [70]. In addition, for another gene *FAT4*, which is also detected by our model but not curated in benchmarking lists, the high prevalence of mutations in *FAT4* are also recognized among the colorectal cancer patients [70]. Gene *GRIN2A* (Glutamate Ionotropic Receptor NMDA Type Subunit 2A) and *POLE* (DNA polymerase epsilon catalytic subunit) are not curated in the 537 CGC genes either. Still, these two genes are detected by our model as top ranked genes in COADREAD results. Recently, *GRIN2A* have been identified as a novel hub driver gene for the stage-II progression of colon adenocarcinoma [71]. Meanwhile, mutations in *POLE* has been reported to be associated with lesions in colon and rectum, and novel mutations in *POLE* detected by exome sequencing also seem to explain the cancer predisposition in colorectal cancer [72]. Moreover, missense mutations in the polymerase genes *POLE* have been identified as rare cause of multiple colorectal adenomas and carcinomas in another recent study [73].

For BRCA results, several genes not included in the benchmarking lists are also detected as top ranked genes by our model. For example, gene *SPEN* is detected by our model from BRCA dataset, which is reported to be capable of regulating tumor growth and cell proliferation [74]. Moreover, nonsense mutations in *SPEN* can also be identified in the ER *α*-expressing breast cancer cell line T47D [74]. Gene *USH2A* is another genes in BRCA results of our model, and *USH2A* mutations have been identified highlighting the molecular diversity observed in triple-negative breast cancers by a recent research [75]. The *OBSCN* is also detected in BRCA results by our model, which is likely to regulate breast cancer progression and metastasis and the prognostic molecular signatures [76].

## Discussion

Discovery mutated driver genes from passenger mutations is one of the primary task in tumorigenesis, and many previous methods find driver genes from somatic mutation data by using interaction network as prior information. In addition to mutation data and network data, mRNA expression patterns of genes are also proven to be highly associated with cancer progressions, which have been widely used in predictions of patients’ clinical outcome and biomarkers of cancers. However, the prior information from mRNA expression data are not exploited by the previous network-based methods. To discover mutated driver genes, we proposed a robust and sparse co-regularized matrix factorization framework, which can effectively incorporate prior information from both interaction network and mRNA expression patterns. Through this framework, we can prioritize the driver gene candidates by their scores in latent representations. To incorporate prior information from mRNA expression and network, graph co-regularization is used in matrix factorization framework to regularize the latent representations of samples and genes with tumor similarity and interaction network. We also use Frobenius norm regularization to prevent overfitting issue. The sparsity-inducing penalty is also used to obtain sparse representations of mutated genes.

When our method is evaluated by two lists of benchmarking genes, our results outperform the results of the framework without at least a portion of the prior information, indicating the contribution of prior information to the performance of driver gene discovery. Furthermore, the detection performance of our methods are largely elevated when compared with the performance of the previous published methods. Statistical test also show that the top scored genes of our methods are significantly different from random selections of the known benchmarking genes. Moreover, while we can find considerable concordance between our method and the other existing methods, our proposed method also discover some important driver genes that are not included in the results of the other methods. The functional enrichment analysis also suggests that the driver candidates discovered by our proposed method are significantly enriched for many well-known cancer related pathways.

Since iRefIndex network [23] is not used as the network information in the original MUFFINN paper, we further rerun the comparison methods with their optimal input information provided in their related papers for evaluation, where iRefIndex is used in HotNet2 as network information [18] and String v10 [77] is used in both MUFFINN and ReMIC [19, 21]. By comparing the results of the competitors with their optimal input information, we find that our model still give the best performance among these methods (Additional file 1: Figures S8). The results also indicate that our model are less sensitive to the choice of prior network information. Notably, we find that the performance of MUFFINN largely increases when the network information changes. Consequently, it is worth using prior network information from several different sources and combining the detection results of both our model and the existing approaches, which can maximize the recognition of driver gene candidates.

Despite the success achieved by our proposed method, some questions are still required for further investigation. A limitation of this study is that the consideration of only the simplified binary mutation matrix can led to a bias with respect to gene lengths. For example, *TTN* is predicted as the third BRCA gene due to its long length, but it is not a cancer gene and therefore this is a false positive prediction. Similar biases are also noticeable in the results of the other cancers. Therefore, how to address the challenge of incorporating mutation rate/types into our method is considered as potential future improvement of our work. Another limitation is that our work encodes the expression similarity and gene-gene interaction as constant matrices, which cannot reflect the dynamic and heterogeneous nature of the expressions and the interactions. In this study, we encode the presence of a somatic mutation on a gene as either 0 or 1 in the matrix by following previous studies [19, 32, 55]. When more than one somatic mutation is incorporated in one gene, the binary encoding strategy may underestimate > 1 somatic mutations on the gene. In comparison, encoding strategy that can incorporate > 1 somatic mutations would be more useful, which are also considered as future work of our study. Moveover, in this study, we use the cutoff of the top 200 genes for the recognition results by following previous work [22]. Note that using a significance threshold like *p*-value can better serve the users. However, how to apply significance test on the results of nonnegative matrix factorization with regularizations is still a challenge, and we plan to address this problem in our future work. Although we have used both somatic mutations and mRNA expressions of genes in our approach, there are also information related to tumor progressions from some other omics, such as copy number alternations and DNA methylation [63]. Since more tumor samples can offer a more comprehensive analysis on tumorigenesis, our future work can also combine the samples of numerous types of cancers to discover driver genes across different cancers [18]. Another possible expansion to our approach is to use some nonlinear loss functions to mining the nonlinearity of the representations of genes [41].

## Conclusions

In summary, we propose a robust and sparse co-regularized nonnegative matrix factorization framework to discover mutated driver genes. This framework can effectively incorporate prior information from both mRNA expression patterns and interaction network of genes. Furthermore, the regularization of robustness and sparseness are also considered in our method. Through evaluation of known benchmarking genes, our method yields better performance compared to NMF framework with at least one of two kinds of the prior information removed. Moreover, our proposed method also outperforms the existing network-based methods, and capture some driver genes missed by the competing methods. In addition, the pathways for which our results are enriched, are highly corresponding to cancer progressions. We hope that our approach can well serve as a driver gene discovery method by offering a comprehensive and sophisticated view of cancer genome.

## Additional file

Additional file 1 **Supplementary figures and tables**. **Figure S1.** The AUCs of precision recall curves of our proposed method when the number of dimensions *K* increases. **Figure S2.** Performance comparison of our proposed method and existing network-based methods, evaluated by IntOGen list. **Figure S3.** Performance of our proposed method when the parameters for sparseness (or robustness) are fixed and the parameters for prior knowledge varies, where *λ*_*RV*_, *λ*_*LV*_ and *λ*_*RU*_ are fixed and *λ*_*LU*_ varies. **Figure S4.** Performance of our proposed method when the parameters for sparseness (or robustness) are fixed and the parameters for prior knowledge varies, where *λ*_*RU*_, *λ*_*LU*_ and *λ*_*RV*_ are fixed and *λ*_*LV*_ varies. **Figure S5.** Performance comparison of our proposed method and existing network-based methods, applied on GBM, COADREAD and BRCA datasets and evaluated by IntOGen list. **Figure S6.** Performance comparison of our proposed method and existing network-based methods, applied on KIRC, THCA and PRAD datasets and evaluated by CGC list. **Figure S7.** Performance comparison of our proposed method and existing network-based methods, applied on KIRC, THCA and PRAD datasets and evaluated by IntOGen list. **Figure S8.** Performance comparison of our proposed method and existing network-based methods with network information from both iRefIndex and String v10. **Table S1.** Fisher’s exact test on the top scored candidates of BRCA results for CGC and IntOGen benchmarking genes. **Table S2.** Fisher’s exact test on the top scored candidates of GBM results for CGC and IntOGen benchmarking genes. **Table S3.** The full list of the top 200 genes detected by our model on GBM dataset. **Table S4.** The full list of the top 200 genes detected by our model on COADREAD dataset. **Table S5.** The full list of the top 200 genes detected by our model on BRCA dataset. **Table S6.** Functional enrichment analysis results for KEGG pathways of the top 200 genes of the proposed method on COADREAD dataset. **Table S7.** Functional enrichment analysis results for KEGG pathways of the top 200 genes of the proposed method on BRCA dataset. (PDF 5670kb)

## Abbreviations

AUC: Area under the curve
BRCA: breast cancer
CGC: Cancer gene census
COADREAD: colon and rectal cancer
CRNMF: co-regularized NMF
FN: False negative
FP: False positive
ICGC: International cancer genome consortium
IntOGen: Integrative onco genomics
GBM: glioblastoma multiforme
KEGG: Kyoto encyclopedia of genes and genomes
NGS: Next generation sequencing
NMF: nonnegative matrix factorization
TCGA: The cancer genome atlas
TP: True positive
