# Supplementary material for: Discovering mutated driver genes through a robust and sparse co-regularized matrix factorization framework with prior information from mRNA expression patterns and interaction network
Source: BMC Bioinformatics. 2018 Jun 5;19:214. doi: 10.1186/s12859-018-2218-y (PMC5989443; doi:10.1186/s12859-018-2218-y)
Supplement: Supplementary file 1 — Supplementary figures and tables. Figure S1. The AUCs of precision recall curves of our proposed method when the number of dimensions K increases. Figure S2. Performance comparison of our proposed method and existing network-based methods, evaluated by IntOGen list. Figure S3. Performance of our proposed method when the parameters for sparseness (or robustness) are fixed and the parameters for prior knowledge varies, where λRV, λLV and λRU are fixed and λLU varies. Figure S4. Performance of our proposed method when the parameters for sparseness (or robustness) are fixed and the parameters for prior knowledge varies, where λRU, λLU and λRV are fixed and λLV varies. Figure S5. Performance comparison of our proposed method and existing network-based methods, applied on GBM, COADREAD and BRCA datasets and evaluated by IntOGen list. Figure S6. Performance comparison of our proposed method and existing network-based methods, applied on KIRC, THCA and PRAD datasets and evaluated by CGC list. Figure S7. Performance comparison of our proposed method and existing network-based methods, applied on KIRC, THCA and PRAD datasets and evaluated by IntOGen list. Figure S8. Performance comparison of our proposed method and existing network-based methods with network information from both iRefIndex and String v10. Table S1. Fisher’s exact test on the top scored candidates of BRCA results for CGC and IntOGen benchmarking genes. Table S2. Fisher’s exact test on the top scored candidates of GBM results for CGC and IntOGen benchmarking genes. Table S3. The full list of the top 200 genes detected by our model on GBM dataset. Table S4. The full list of the top 200 genes detected by our model on COADREAD dataset. Table S5. The full list of the top 200 genes detected by our model on BRCA dataset. Table S6. Functional enrichment analysis results for KEGG pathways of the top 200 genes of the proposed method on COADREAD dataset. Table S7. Functional enrichment analysis results for KEGG pathways [file 12859_2018_2218_MOESM1_ESM.pdf]

Additional file 1 — Supplementary Figures and Tables

Discovering mutated driver genes through a robust and sparse co-regularized matrix factorization framework with prior information from mRNA expression patterns and interaction network

Jianing Xi[1], Minghui Wang[1,2*], Ao Li[1,2]

[1]School of Information Science and Technology, University of Science and Technology of China, Hefei 230027, PR China
[2]Centers for Biomedical Engineering, University of Science and Technology of China, Hefei 230027, PR China
*Corresponding authors: mhwang@ustc.edu.cn

List of Figures

Figure S1 The AUCs of precision recall curves of our proposed method when the number of dimensions K increases, evaluated by (a)-(c) CGC [1] and (d)-(f) IntOGen lists [2]. The results are for datatsets of (a) (d) GBM, (b) (e) COADREAD and (c) (f) BRCA. . . . 3

Figure S2 Performance comparison of NMF with different kinds of prior information, evaluated by IntOGen list [2]. The precision recall curves of the results of our proposed method ("Proposed": red), NMF with information of mRNA expression pattern similarity ("Only expression": orange), NMF with only network information ("Only network": yellow), and NMF with no prior information ("No prior": dark red), for datatsets of (a) GBM, (b) COADREAD and (c) BRCA. The AUCs of precision recall curves of "Proposed", "Only expression", "Only network" and "No prior", displayed as bar plot, for datatsets of (d) GBM, (e) COADREAD and (f) BRCA. . . . 3

Figure S3 Performance of our proposed method when the parameters for sparseness (or robustness) are fixed and the parameters for prior knowledge varies, where λ_{RV}, λ_{LV} and λ_{RU} are fixed to 1.0 and λ_{LU} varies in range of 0, 0.001, 0.01, 0.1 and 1, evaluated by (a)-(f) CGC [1] and (g)-(l) IntOGen lists [2]. The precision recall curves of the results of our methods for datatsets of (a) (g) GBM, (b) (h) COADREAD and (c) (i) BRCA. The AUCs of precision recall curves displayed as bar plot, for datatsets of (d) (j) GBM, (e) (k) COADREAD and (f) (l) BRCA. 4

Figure S4 Performance of our proposed method when the parameters for sparseness (or robustness) are fixed and the parameters for prior knowledge varies, where λ_{RU}, λ_{LU} and λ_{RV} are fixed to 1.0 and λ_{LV} varies in range of 0, 0.001, 0.01, 0.1 and 1, evaluated by (a)-(f) CGC [1] and (g)-(l) IntOGen lists [2]. The precision recall curves of the results of our methods for datatsets of (a) (g) GBM, (b) (h) COADREAD and (c) (i) BRCA. The AUCs of precision recall curves displayed as bar plot, for datatsets of (d) (j) GBM, (e) (k) COADREAD and (f) (l) BRCA. 5

Figure S5 Performance comparison of our proposed method and existing network-based methods, evaluated by IntOGen list [2]. The precision recall curves of the results of our proposed method (red), DawnRank (brown), DriverNet (medium purple), HotNet2 (orange), ReMIC(β=0.01) (green), ReMIC(β=0.02) (cyan), ReMIC(β=0.03) (blue), MUFFINN(DNmax) (violet), MUFFINN(DNsum) (magenta) and baseline by mutation frequency (gray), for datatsets of (a) GBM, (b) COADREAD and (c) BRCA. The AUCs of precision recall curves of the competing methods, displayed as bar plot, for datatsets of (d) GBM, (e) COADREAD and (f) BRCA. The black dash lines in (d)-(f) represent the AUC values of baseline by mutation frequency. . . . 6

Figure S6 Performance comparison of our proposed method and existing network-based methods, evaluated by CGC list [1]. The precision recall curves of the results of our proposed method (red), DawnRank (brown), DriverNet (medium purple), HotNet2 (orange), ReMIC(β=0.01) (green), ReMIC(β=0.02) (cyan), ReMIC(β=0.03) (blue), MUFFINN(DNmax) (violet), MUFFINN(DNsum) (magenta) and baseline by mutation frequency (gray), for datatsets of (a) KIRC, (b) THCA and (c) PRAD. The AUCs of precision recall curves of the competing methods, displayed as bar plot, for datatsets of (d) KIRC, (e) THCA and (f) PRAD. The black dash lines in (d)-(f) represent the AUC values of baseline by mutation frequency. . . . 7

Figure S7 Performance comparison of our proposed method and existing network-based methods, evaluated by IntOGen list [2]. The precision recall curves of the results of our proposed method (red), DawnRank (brown), DriverNet (medium purple), HotNet2 (orange), ReMIC(β=0.01) (green), ReMIC(β=0.02) (cyan), ReMIC(β=0.03) (blue), MUFFINN(DNmax) (violet), MUFFINN(DNsum) (magenta) and baseline by mutation frequency (gray), for datatsets of (a) KIRC, (b) THCA and (c) PRAD. The AUCs of precision recall curves of the competing methods, displayed as bar plot, for datatsets of (d) KIRC, (e) THCA and (f) PRAD. The black dash lines in (d)-(f) represent the AUC values of baseline by mutation frequency. . . . 8

Figure S8 Performance comparison of our proposed method and existing network-based methods with network information from both iRefIndex [3] and String v10 [4], evaluated by (a)-(f) CGC [1] and (g)-(l) IntOGen list [2]. The precision recall curves of the results of our proposed method for iRefIndex (red dashed line) and String v10 (red dotted line), HotNet2 for iRefIndex (orange dashed line) and String v10 (orange dotted line), ReMIC(β=0.01) for iRefIndex (green dashed line) and String v10 (green dotted line), ReMIC(β=0.02) for iRefIndex (cyan dashed line) and String v10 (cyan dotted line), ReMIC(β=0.03) for iRefIndex (blue dashed line) and String v10 (blue dotted line), MUFFINN(DNmax) for iRefIndex (violet dashed line) and String v10 (violet dotted line), MUFFINN(DNsum) for iRefIndex (magenta dashed line) and String v10 (magenta dotted line), for datatsets of (a) (g) KIRC, (b) (h) THCA and (c) (i) PRAD. The AUCs of precision recall curves of the competing methods, displayed as bar plot, for datatsets of (d) (j) KIRC, (e) (k) THCA and (f) (l) PRAD. 9

List of Tables

Table S1 Fisher's exact test on the top scored candidates of GBM results for CGC and IntOGen benchmarking genes. The p-values are for the results our proposed method, HotNet2, ReMIC(β=0.01), ReMIC(β=0.02), ReMIC(β=0.03), MUFFINN(DNmax) and MUFFINN(DNsum). 10

Table S2 Fisher's exact test on the top scored candidates of BRCA results for CGC and IntOGen benchmarking genes. The p-values are for the results our proposed method, HotNet2, ReMIC(β=0.01), ReMIC(β=0.02), ReMIC(β=0.03), MUFFINN(DNmax) and MUFFINN(DNsum). 10

Table S3 The full list of the top 200 genes detected by our model on GBM dataset. The table demonstrates the ranks of the genes, gene symbols, mutation rates, the included benchmarking gene lists [1, 2] of the genes and the other methods [5, 6, 7] by which the genes are also detected. 11

Table S4 The full list of the top 200 genes detected by our model on COADREAD dataset. The table demonstrates the ranks of the genes, gene symbols, mutation rates, the included benchmarking gene lists [1, 2] of the genes and the other methods [5, 6, 7] by which the genes are also detected. 14

Table S5 The full list of the top 200 genes detected by our model on BRCA dataset. The table demonstrates the ranks of the genes, gene symbols, mutation rates, the included benchmarking gene lists [1, 2] of the genes and the other methods [5, 6, 7] by which the genes are also detected. 17

Table S6 Functional enrichment analysis results for KEGG pathways [8] of the top 200 genes of the proposed method on COADREAD dataset. The pathways are sorted by their enrichment p-values. 20

Table S7 Functional enrichment analysis results for KEGG pathways [8] of the top 200 genes of the proposed method on BRCA dataset. The pathways are sorted by their enrichment p-values. 20

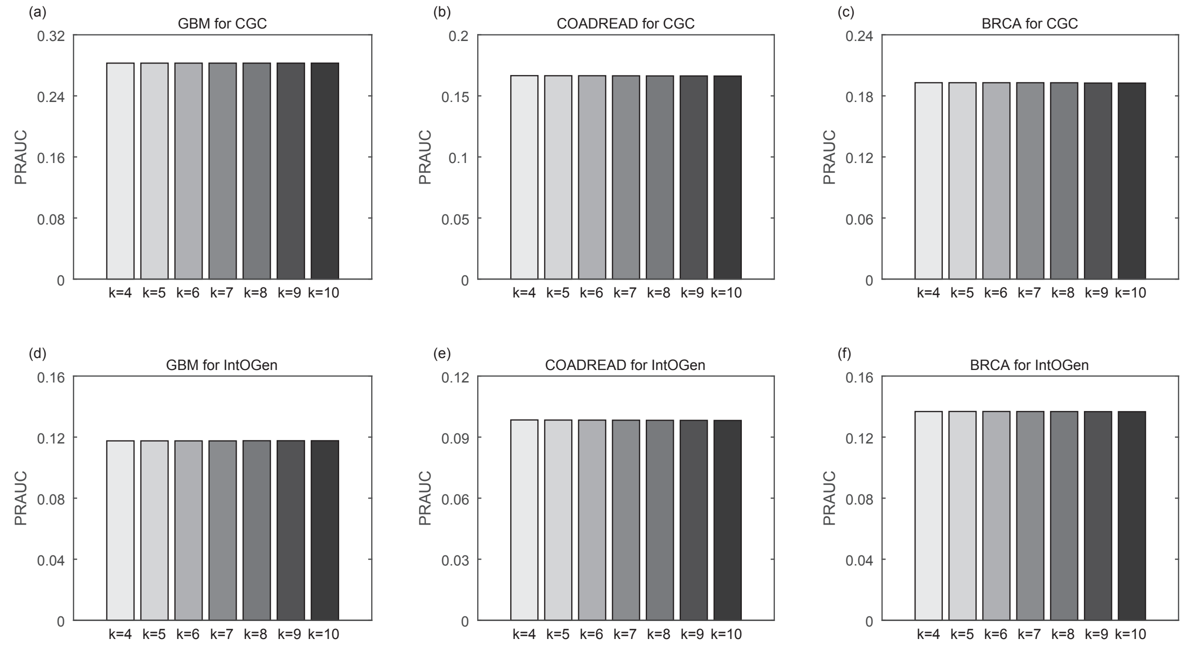

Figure S1. The AUCs of precision recall curves of our proposed method when the number of dimensions K increases, evaluated by (a)-(c) CGC [1] and (d)-(f) IntOGen lists [2]. The results are for datatsets of (a) (d) GBM, (b) (e) COADREAD and (c) (f) BRCA.

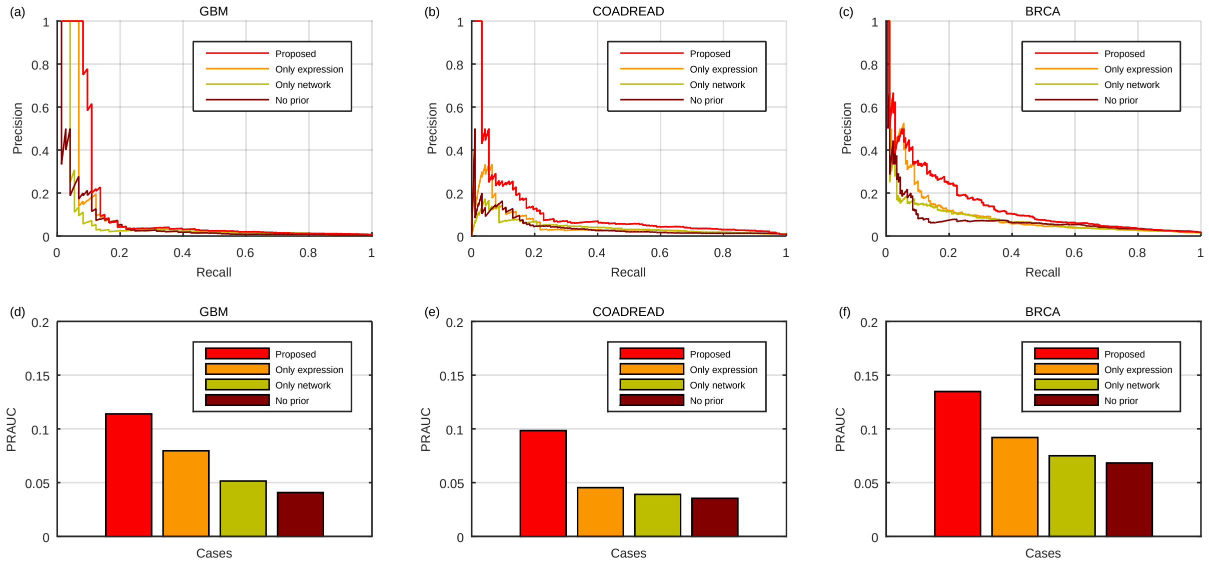

Figure S2. Performance comparison of NMF with different kinds of prior information, evaluated by IntOGen list [2]. The precision recall curves of the results of our proposed method ("Proposed": red), NMF with information of mRNA expression pattern similarity ("Only expression": orange), NMF with only network information ("Only network": yellow), and NMF with no prior information ("No prior": dark red), for datatsets of (a) GBM, (b) COADREAD and (c) BRCA. The AUCs of precision recall curves of "Proposed", "Only expression", "Only network" and "No prior", displayed as bar plot, for datatsets of (d) GBM, (e) COADREAD and (f) BRCA.

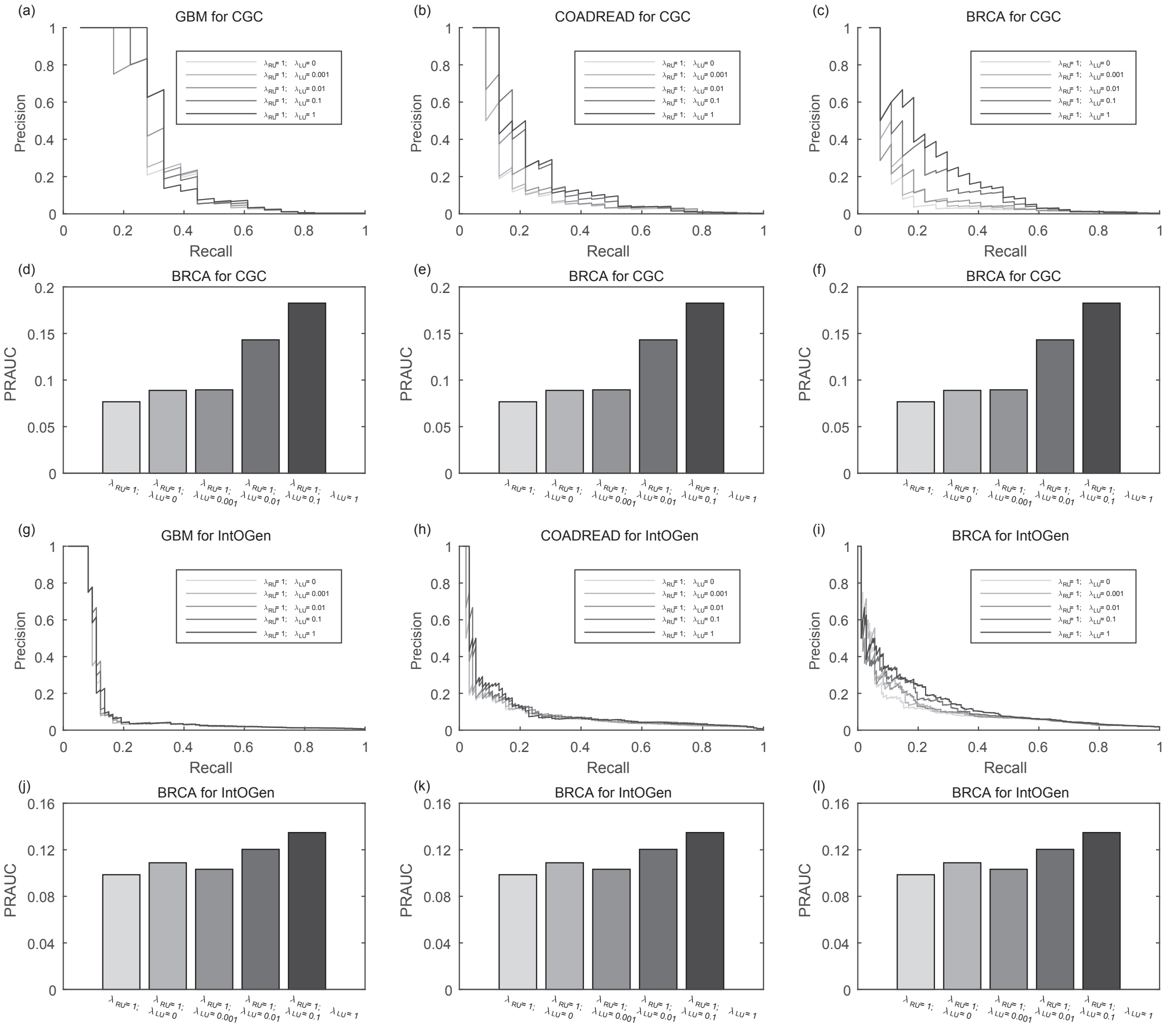

Figure S3. Performance of our proposed method when the parameters for sparseness (or robustness) are fixed and the parameters for prior knowledge varies, where λ_{RV}, λ_{LV} and λ_{RU} are fixed to 1.0 and λ_{LU} varies in range of 0, 0.001, 0.01, 0.1 and 1, evaluated by (a)-(f) CGC [1] and (g)-(l) IntOGen lists [2]. The precision recall curves of the results of our methods for datatsets of (a) (g) GBM, (b) (h) COADREAD and (c) (i) BRCA. The AUCs of precision recall curves displayed as bar plot, for datatsets of (d) (j) GBM, (e) (k) COADREAD and (f) (l) BRCA.

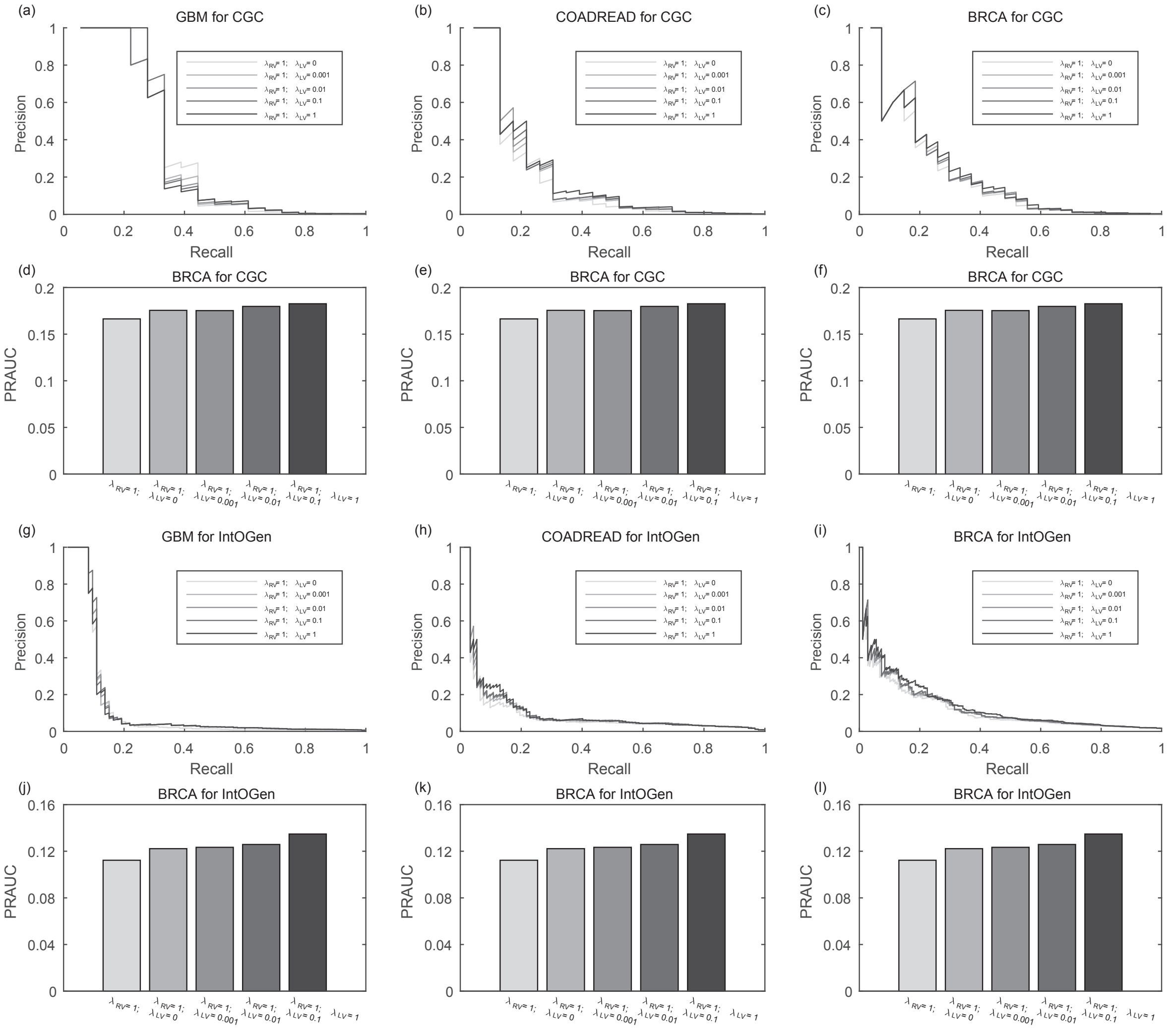

Figure S4. Performance of our proposed method when the parameters for sparseness (or robustness) are fixed and the parameters for prior knowledge varies, where λ_{RU}, λ_{LU} and λ_{RV} are fixed to 1.0 and λ_{LV} varies in range of 0, 0.001, 0.01, 0.1 and 1, evaluated by (a)-(f) CGC [1] and (g)-(l) IntOGen lists [2]. The precision recall curves of the results of our methods for datatsets of (a) (g) GBM, (b) (h) COADREAD and (c) (i) BRCA. The AUCs of precision recall curves displayed as bar plot, for datatsets of (d) (j) GBM, (e) (k) COADREAD and (f) (l) BRCA.

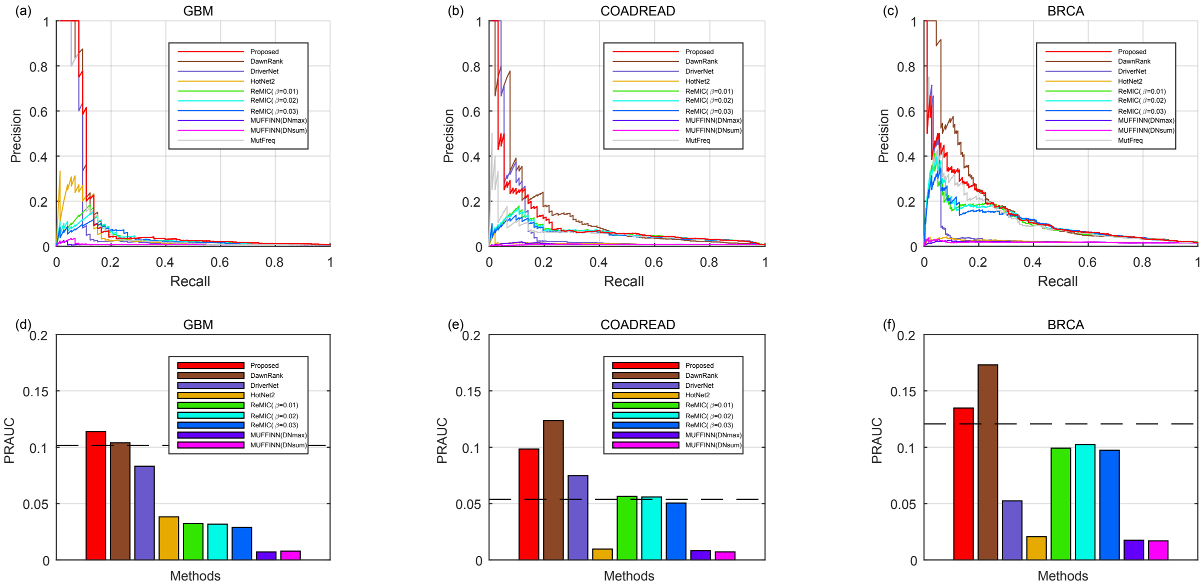

Figure S5. Performance comparison of our proposed method and existing network-based methods, evaluated by IntOGen list [2]. The precision recall curves of the results of our proposed method (red), DawnRank (brown), DriverNet (medium purple), HotNet2 (orange), ReMIC(β=0.01) (green), ReMIC(β=0.02) (cyan), ReMIC(β=0.03) (blue), MUFFINN(DNmax) (violet), MUFFINN(DNsum) (magenta) and baseline by mutation frequency (gray), for datatsets of (a) GBM, (b) COADREAD and (c) BRCA. The AUCs of precision recall curves of the competing methods, displayed as bar plot, for datatsets of (d) GBM, (e) COADREAD and (f) BRCA. The black dash lines in (d)-(f) represent the AUC values of baseline by mutation frequency.

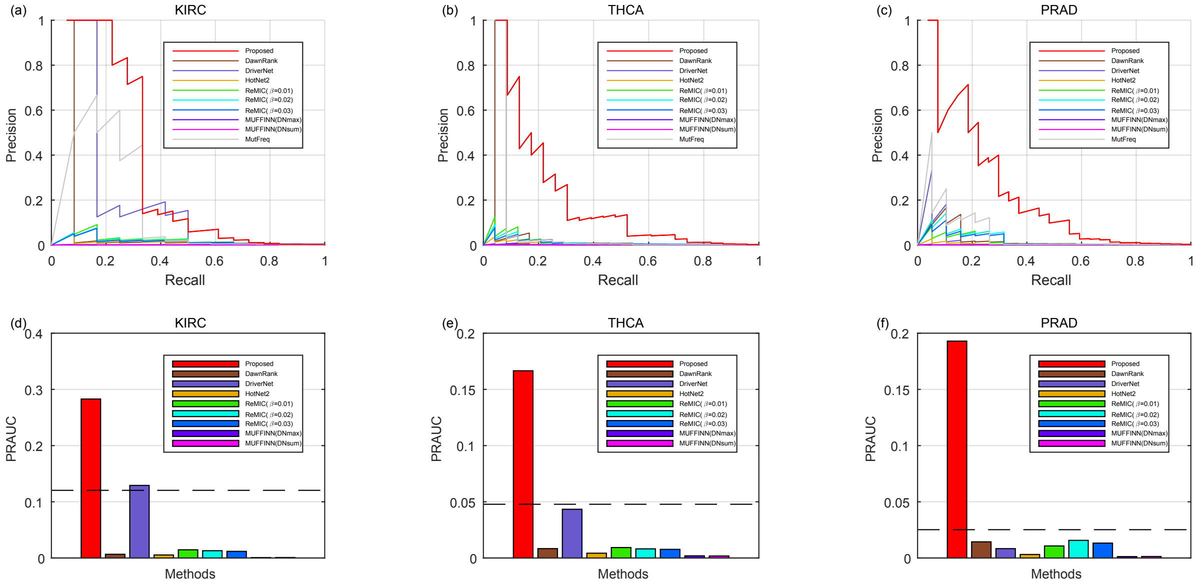

Figure S6. Performance comparison of our proposed method and existing network-based methods, evaluated by CGC list [1]. The precision recall curves of the results of our proposed method (red), DawnRank (brown), DriverNet (medium purple), HotNet2 (orange), ReMIC(β=0.01) (green), ReMIC(β=0.02) (cyan), ReMIC(β=0.03) (blue), MUFFINN(DNmax) (violet), MUFFINN(DNsum) (magenta) and baseline by mutation frequency (gray), for datatsets of (a) KIRC, (b) THCA and (c) PRAD. The AUCs of precision recall curves of the competing methods, displayed as bar plot, for datatsets of (d) KIRC, (e) THCA and (f) PRAD. The black dash lines in (d)-(f) represent the AUC values of baseline by mutation frequency.

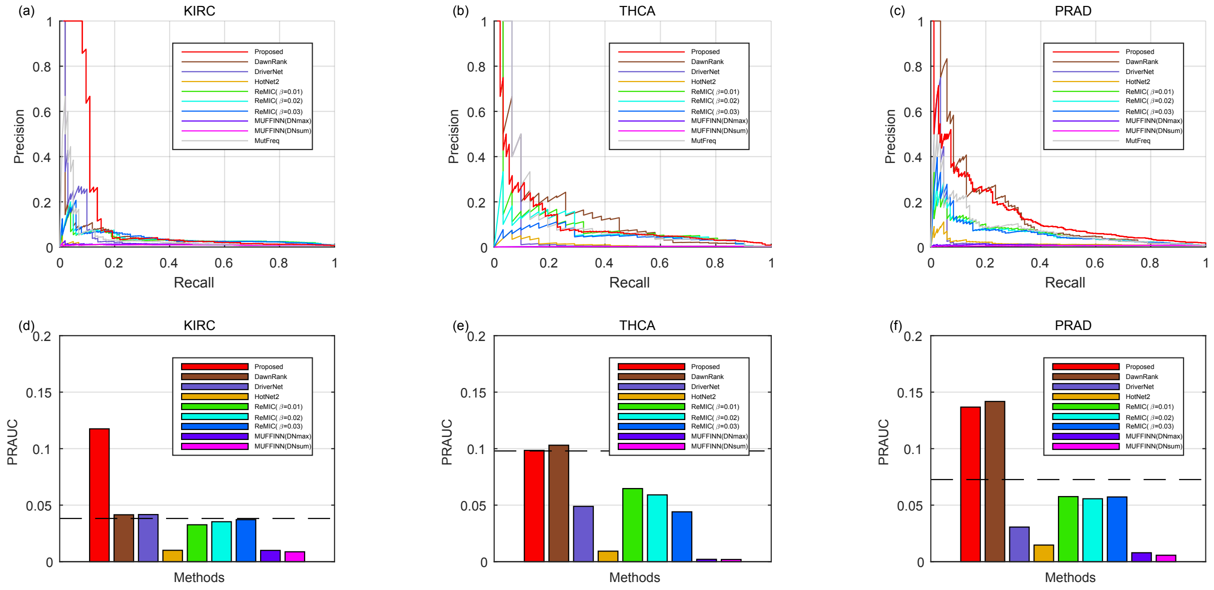

Figure S7. Performance comparison of our proposed method and existing network-based methods, evaluated by IntOGen list [2]. The precision recall curves of the results of our proposed method (red), DawnRank (brown), DriverNet (medium purple), HotNet2 (orange), ReMIC(β=0.01) (green), ReMIC(β=0.02) (cyan), ReMIC(β=0.03) (blue), MUFFINN(DNmax) (violet), MUFFINN(DNsum) (magenta) and baseline by mutation frequency (gray), for datatsets of (a) KIRC, (b) THCA and (c) PRAD. The AUCs of precision recall curves of the competing methods, displayed as bar plot, for datatsets of (d) KIRC, (e) THCA and (f) PRAD. The black dash lines in (d)-(f) represent the AUC values of baseline by mutation frequency.

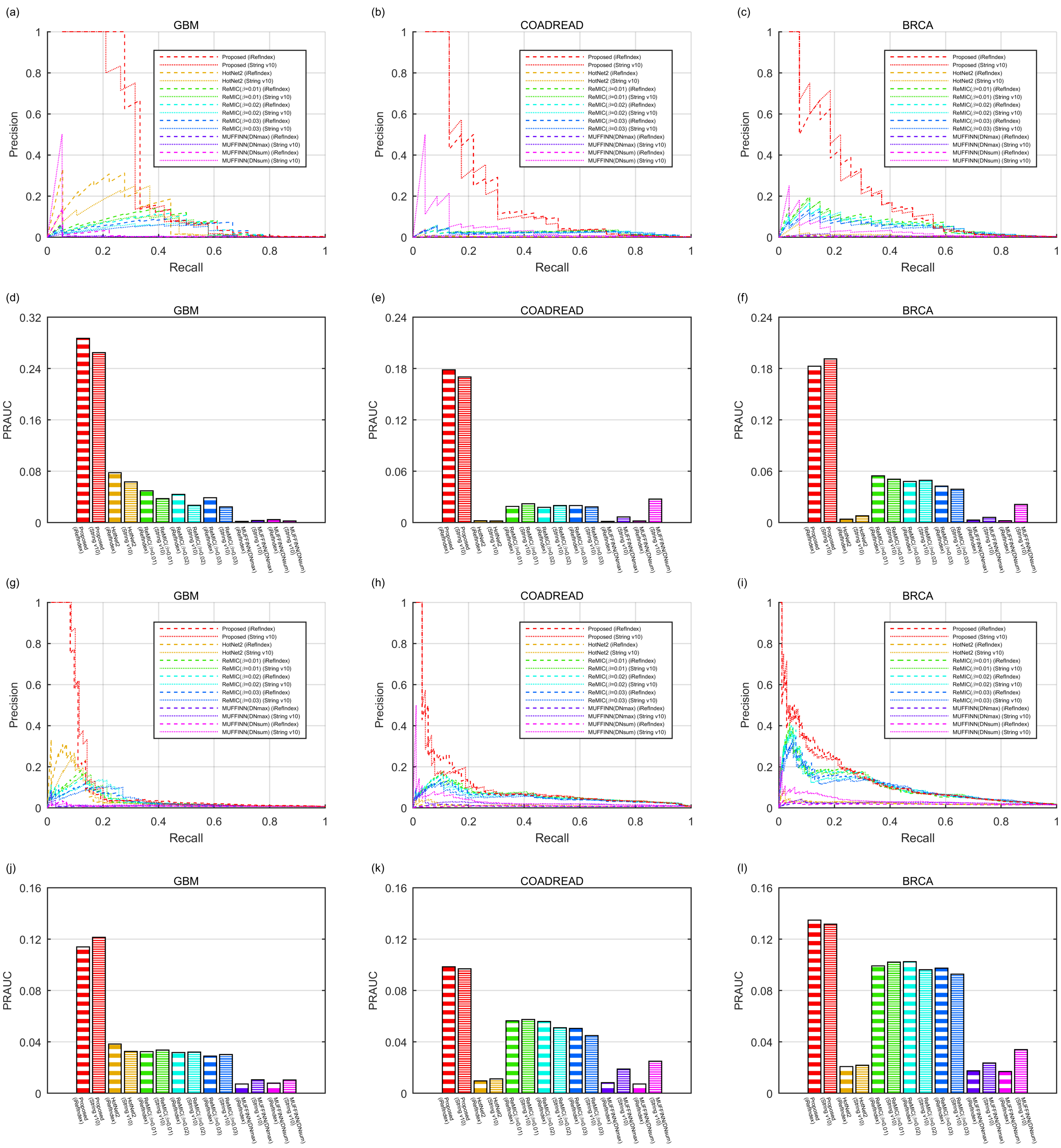

Figure S8. Performance comparison of our proposed method and existing network-based methods with network information from both iRefIndex [3] and String v10 [4], evaluated by (a)-(f) CGC [1] and (g)-(l) IntOGen list [2]. The precision recall curves of the results of our proposed method for iRefIndex (red dashed line) and String v10 (red dotted line), HotNet2 for iRefIndex (orange dashed line) and String v10 (orange dotted line), ReMIC(β=0.01) for iRefIndex (green dashed line) and String v10 (green dotted line), ReMIC(β=0.02) for iRefIndex (cyan dashed line) and String v10 (cyan dotted line), ReMIC(β=0.03) for iRefIndex (blue dashed line) and String v10 (blue dotted line), MUFFINN(DNmax) for iRefIndex (violet dashed line) and String v10 (violet dotted line), MUFFINN(DNsum) for iRefIndex (magenta dashed line) and String v10 (magenta dotted line), for datatsets of (a) (g) KIRC, (b) (h) THCA and (c) (i) PRAD. The AUCs of precision recall curves of the competing methods, displayed as bar plot, for datatsets of (d) (j) KIRC, (e) (k) THCA and (f) (l) PRAD.

Table S1. Fisher's exact test on the top scored candidates of GBM results for CGC and IntOGen benchmarking genes. The p-values are for the results our proposed method, HotNet2, ReMIC(β=0.01), ReMIC(β=0.02), ReMIC(β=0.03), MUFFINN(DNmax) and MUFFINN(DNsum).

	CGC				IntOGen			
Top	50	100	150	200	50	100	150	200
Proposed	4.02e-13	6.57e-13	2.49e-15	5.35e-16	2.79e-13	3.71e-10	7.90e-11	1.63e-10
HotNet2	1.97e-15	6.57e-13	1.79e-11	1.81e-10	2.79e-13	3.71e-10	1.32e-09	2.71e-08
ReMIC($\beta = 0.01$)	4.02e-13	5.57e-15	2.11e-17	5.35e-16	1.28e-11	1.59e-11	4.27e-12	1.10e-11
ReMIC($\beta = 0.02$)	5.15e-05	5.57e-15	2.11e-17	5.35e-16	2.26e-04	1.59e-11	4.27e-12	1.10e-11
ReMIC($\beta = 0.03$)	7.17e-02	6.57e-13	2.35e-13	4.94e-18	3.35e-03	3.71e-10	4.27e-12	1.10e-11
MUFFINN(DNmax)	7.17e-02	1.39e-01	2.01e-01	2.59e-01	2.61e-01	1.22e-01	6.16e-02	1.19e-01
MUFFINN(DNmax)	7.17e-02	1.39e-01	6.01e-05	1.83e-04	2.61e-01	1.22e-01	2.07e-03	7.04e-03

Table S2. Fisher's exact test on the top scored candidates of BRCA results for CGC and IntOGen benchmarking genes. The p-values are for the results our proposed method, HotNet2, ReMIC(β=0.01), ReMIC(β=0.02), ReMIC(β=0.03), MUFFINN(DNmax) and MUFFINN(DNsum).

	CGC				IntOGen			
Top	50	100	150	200	50	100	150	200
Proposed	4.35e-18	6.62e-21	1.61e-18	1.56e-20	2.03e-19	1.06e-28	3.79e-34	1.44e-34
HotNet2	1.00e-00	2.01e-01	2.86e-01	3.62e-01	5.23e-01	5.95e-02	2.86e-01	2.22e-01
ReMIC($\beta = 0.01$)	7.31e-08	5.06e-13	7.86e-15	1.16e-18	5.14e-12	7.26e-14	3.93e-22	1.21e-30
ReMIC($\beta = 0.02$)	7.31e-08	5.06e-13	7.86e-15	7.38e-17	7.60e-15	1.42e-17	3.93e-22	7.07e-27
ReMIC($\beta = 0.03$)	7.31e-08	3.13e-11	2.06e-11	1.93e-13	2.09e-13	7.15e-19	2.39e-17	1.86e-24
MUFFINN(DNmax)	1.00e-00	1.00e-00	1.00e-00	1.00e-00	1.00e-00	1.00e-00	7.29e-01	1.00e-00
MUFFINN(DNmax)	1.00e-00	1.00e-00	1.00e-00	1.00e-00	5.23e-01	1.82e-01	4.86e-01	5.42e-01

Table S3. The full list of the top 200 genes detected by our model on GBM dataset. The table demonstrates the ranks of the genes, gene symbols, mutation rates, the included benchmarking gene lists [1, 2] of the genes and the other methods [5, 6, 7] by which the genes are also detected.

Rank	Symbol	MutRate	Benchmarking	OtherMethod	Rank	Symbol	MutRate	Benchmarking	OtherMethod
1	TP53	37.8%	CGC; IntOGen	HotNet2; ReMIC(β=0.01); ReMIC(β=0.02); ReMIC(β=0.03)	101	PROX1	1.2%		ReMIC(β=0.01); ReMIC(β=0.02); MUFFINN(DNsum)
2	PTEN	35.4%	CGC; IntOGen	HotNet2; ReMIC(β=0.01); ReMIC(β=0.02); ReMIC(β=0.03)	102	AXL	1.2%		HotNet2; ReMIC(β=0.01); ReMIC(β=0.02); ReMIC(β=0.03); MUFFINN(DNmax); MUFFINN(DNsum)
3	EGFR	18.3%	CGC; IntOGen	HotNet2; ReMIC(β=0.01); ReMIC(β=0.02); ReMIC(β=0.03)	103	PTCH1	1.2%		HotNet2; ReMIC(β=0.01); ReMIC(β=0.02); MUFFINN(DNsum)
4	NF1	15.9%	CGC; IntOGen	ReMIC(β=0.01); ReMIC(β=0.02); ReMIC(β=0.03)	104	PML	1.2%		ReMIC(β=0.01); ReMIC(β=0.02); ReMIC(β=0.03); MUFFINN(DNsum)
5	PIK3R1	11.0%	CGC; IntOGen	HotNet2; ReMIC(β=0.01); ReMIC(β=0.02); ReMIC(β=0.03)	105	MTOR	1.2%		ReMIC(β=0.01); ReMIC(β=0.02); ReMIC(β=0.03); MUFFINN(DNsum)
6	RB1	11.0%	IntOGen	HotNet2; ReMIC(β=0.01); ReMIC(β=0.02); ReMIC(β=0.03)	106	ADAM12	1.2%		ReMIC(β=0.01); ReMIC(β=0.02); MUFFINN(DNmax); MUFFINN(DNsum)
7	ERBB2	8.5%		HotNet2; ReMIC(β=0.01); ReMIC(β=0.02); ReMIC(β=0.03)	107	MAPK13	1.2%		ReMIC(β=0.01); ReMIC(β=0.02); ReMIC(β=0.03); MUFFINN(DNsum)
8	DST	7.3%		ReMIC(β=0.01); ReMIC(β=0.02); ReMIC(β=0.03)	108	AURKB	1.2%		HotNet2; ReMIC(β=0.01); ReMIC(β=0.02); MUFFINN(DNmax); MUFFINN(DNsum)
9	PIK3CA	7.3%	CGC; IntOGen	HotNet2; ReMIC(β=0.01); ReMIC(β=0.02); ReMIC(β=0.03); MUFFINN(DNsum)	109	MDM2	1.2%	CGC	ReMIC(β=0.01); ReMIC(β=0.02); ReMIC(β=0.03); MUFFINN(DNsum)
10	MSH6	4.9%		HotNet2; ReMIC(β=0.01); ReMIC(β=0.02); ReMIC(β=0.03)	110	TGFBR2	1.2%	IntOGen	ReMIC(β=0.01); ReMIC(β=0.02); MUFFINN(DNsum)
11	BCL11A	4.9%		ReMIC(β=0.01); ReMIC(β=0.02); ReMIC(β=0.03); MUFFINN(DNmax)	111	NBN	1.2%		ReMIC(β=0.01); ReMIC(β=0.02); ReMIC(β=0.03); MUFFINN(DNsum)
12	ITGB3	3.7%		HotNet2; ReMIC(β=0.01); ReMIC(β=0.02); ReMIC(β=0.03)	112	CD46	1.2%		MUFFINN(DNmax); MUFFINN(DNsum)
13	FN1	3.7%	IntOGen	HotNet2; ReMIC(β=0.01); ReMIC(β=0.02); ReMIC(β=0.03)	113	RTN1	1.2%		MUFFINN(DNsum)
14	BRCA2	3.7%		HotNet2; ReMIC(β=0.01); ReMIC(β=0.02); ReMIC(β=0.03)	114	ID3	1.2%		ReMIC(β=0.01); ReMIC(β=0.02); MUFFINN(DNsum)
15	CHEK2	3.7%		HotNet2; ReMIC(β=0.01); ReMIC(β=0.02); ReMIC(β=0.03)	115	DGKD	1.2%		ReMIC(β=0.01); MUFFINN(DNsum)
16	EP300	3.7%		ReMIC(β=0.01); ReMIC(β=0.02); ReMIC(β=0.03)	116	CTSH	1.2%		MUFFINN(DNsum)
17	CDKN2A	3.7%		HotNet2; ReMIC(β=0.01); ReMIC(β=0.02); ReMIC(β=0.03)	117	ASPM	1.2%		HotNet2; ReMIC(β=0.01); ReMIC(β=0.02); ReMIC(β=0.03)
18	TNC	3.7%		HotNet2; ReMIC(β=0.01); ReMIC(β=0.02); ReMIC(β=0.03)	118	ARNT	1.2%		HotNet2; ReMIC(β=0.01); ReMIC(β=0.02); ReMIC(β=0.03); MUFFINN(DNmax); MUFFINN(DNsum)
19	TNK2	3.7%		HotNet2; ReMIC(β=0.01); ReMIC(β=0.02); ReMIC(β=0.03)	119	PRKD2	1.2%		ReMIC(β=0.01); ReMIC(β=0.02); ReMIC(β=0.03); MUFFINN(DNmax); MUFFINN(DNsum)
20	FURIN	2.4%		HotNet2; ReMIC(β=0.01); ReMIC(β=0.02); ReMIC(β=0.03)	120	ANXA7	1.2%		ReMIC(β=0.01); MUFFINN(DNmax); MUFFINN(DNsum)
21	PRKCZ	2.4%		ReMIC(β=0.01); ReMIC(β=0.02); ReMIC(β=0.03)	121	TNFRSF1B	1.2%		ReMIC(β=0.01); ReMIC(β=0.02); MUFFINN(DNsum)
22	PDGFRB	2.4%		HotNet2; ReMIC(β=0.01); ReMIC(β=0.02); ReMIC(β=0.03)	122	RINT1	1.2%		ReMIC(β=0.01); MUFFINN(DNsum)
23	ITGB2	2.4%		ReMIC(β=0.01); ReMIC(β=0.02); ReMIC(β=0.03)	123	RUNX1T1	1.2%		ReMIC(β=0.01); ReMIC(β=0.02); ReMIC(β=0.03); MUFFINN(DNsum)
24	HSP90AA1	2.4%		ReMIC(β=0.01); ReMIC(β=0.02); ReMIC(β=0.03)	124	TRIM33	1.2%		ReMIC(β=0.01); ReMIC(β=0.02); MUFFINN(DNsum)
25	TRRAP	2.4%		ReMIC(β=0.01); ReMIC(β=0.02); ReMIC(β=0.03)	125	ST7	1.2%		ReMIC(β=0.01); MUFFINN(DNsum)

26	MLH1	2.4%		ReMIC(β=0.01); ReMIC(β=0.02); ReMIC(β=0.03)	126	CHEK1	1.2%		ReMIC(β=0.01); ReMIC(β=0.02); ReMIC(β=0.03); MUFFINN(DNsum)
27	MAPK9	2.4%		HotNet2; ReMIC(β=0.01); ReMIC(β=0.02); ReMIC(β=0.03)	127	GLI3	1.2%		HotNet2; ReMIC(β=0.01); ReMIC(β=0.02); MUFFINN(DNsum)
28	A2M	2.4%		HotNet2; ReMIC(β=0.01); ReMIC(β=0.02); ReMIC(β=0.03)	128	TIMP2	1.2%		MUFFINN(DNmax); MUFFINN(DNsum)
29	PRKDC	2.4%		ReMIC(β=0.01); ReMIC(β=0.02); ReMIC(β=0.03)	129	MTAP	1.2%		MUFFINN(DNsum)
30	BCR	2.4%		HotNet2; ReMIC(β=0.01); ReMIC(β=0.02); ReMIC(β=0.03)	130	KIT	1.2%		ReMIC(β=0.01); ReMIC(β=0.02); ReMIC(β=0.03); MUFFINN(DNsum)
31	IRS1	2.4%		HotNet2; ReMIC(β=0.01); ReMIC(β=0.02); ReMIC(β=0.03)	131	TSC1	1.2%		ReMIC(β=0.01); ReMIC(β=0.02); ReMIC(β=0.03); MUFFINN(DNsum)
32	ING4	2.4%		HotNet2; ReMIC(β=0.01); ReMIC(β=0.02); ReMIC(β=0.03)	132	ADAM15	1.2%		MUFFINN(DNmax); MUFFINN(DNsum)
33	TAF1	2.4%		HotNet2; ReMIC(β=0.01); ReMIC(β=0.02); ReMIC(β=0.03)	133	DHTKD1	1.2%		MUFFINN(DNsum)
34	FBXW7	2.4%		HotNet2; ReMIC(β=0.01); ReMIC(β=0.02); ReMIC(β=0.03)	134	EPHA2	1.2%		HotNet2; ReMIC(β=0.01); ReMIC(β=0.02); ReMIC(β=0.03); MUFFINN(DNsum)
35	MSH2	2.4%		HotNet2; ReMIC(β=0.01); ReMIC(β=0.02); ReMIC(β=0.03)	135	CASP9	1.2%		ReMIC(β=0.01); ReMIC(β=0.02); ReMIC(β=0.03); MUFFINN(DNsum)
36	NOTCH1	2.4%		HotNet2; ReMIC(β=0.01); ReMIC(β=0.02); ReMIC(β=0.03)	136	DCX	1.2%		ReMIC(β=0.01); MUFFINN(DNsum)
37	ROR2	2.4%		ReMIC(β=0.01); ReMIC(β=0.02); ReMIC(β=0.03)	137	PRKCB	1.2%		ReMIC(β=0.01); ReMIC(β=0.02); ReMIC(β=0.03); MUFFINN(DNsum)
38	CENPF	2.4%		ReMIC(β=0.01); ReMIC(β=0.02); ReMIC(β=0.03)	138	IL1RL1	1.2%		MUFFINN(DNsum)
39	EPHA7	2.4%		ReMIC(β=0.01); ReMIC(β=0.02); ReMIC(β=0.03)	139	DOCK1	1.2%		ReMIC(β=0.01); ReMIC(β=0.02)
40	IQGAP1	2.4%		ReMIC(β=0.01); ReMIC(β=0.02); ReMIC(β=0.03)	140	TRIM24	1.2%		ReMIC(β=0.01); ReMIC(β=0.02); MUFFINN(DNsum)
41	MET	2.4%	IntOGen	HotNet2; ReMIC(β=0.01); ReMIC(β=0.02); ReMIC(β=0.03)	141	PDGFRA	1.2%	CGC	HotNet2; ReMIC(β=0.01); ReMIC(β=0.02); ReMIC(β=0.03); MUFFINN(DNsum)
42	LTF	2.4%		ReMIC(β=0.01); ReMIC(β=0.02); ReMIC(β=0.03)	142	POU2F1	1.2%		ReMIC(β=0.01); ReMIC(β=0.02); MUFFINN(DNsum)
43	BCAR1	2.4%		HotNet2; ReMIC(β=0.01); ReMIC(β=0.02); ReMIC(β=0.03)	143	KRAS	1.2%	IntOGen	HotNet2; ReMIC(β=0.01); ReMIC(β=0.02); ReMIC(β=0.03); MUFFINN(DNsum)
44	PTPN11	2.4%	IntOGen	ReMIC(β=0.01); ReMIC(β=0.02); ReMIC(β=0.03)	144	CNTFR	1.2%		MUFFINN(DNsum)
45	KLF6	2.4%	CGC	HotNet2; ReMIC(β=0.01); ReMIC(β=0.02); ReMIC(β=0.03)	145	PIM1	1.2%		ReMIC(β=0.01); ReMIC(β=0.02); MUFFINN(DNmax); MUFFINN(DNsum)
46	TCF12	2.4%		ReMIC(β=0.01); ReMIC(β=0.02); ReMIC(β=0.03)	146	RPS6KA3	1.2%		ReMIC(β=0.01); MUFFINN(DNsum)
47	GATA3	2.4%		ReMIC(β=0.01); ReMIC(β=0.02); ReMIC(β=0.03)	147	TRIM2	1.2%		MUFFINN(DNsum)
48	COL1A1	2.4%		HotNet2; ReMIC(β=0.01); ReMIC(β=0.02); ReMIC(β=0.03)	148	COL1A2	1.2%		HotNet2; ReMIC(β=0.01); ReMIC(β=0.02); ReMIC(β=0.03); MUFFINN(DNmax); MUFFINN(DNsum)
49	NMBR	2.4%		ReMIC(β=0.01); ReMIC(β=0.02); ReMIC(β=0.03)	149	EPHA4	1.2%		MUFFINN(DNsum)
50	BAI3	2.4%		ReMIC(β=0.01); ReMIC(β=0.02); ReMIC(β=0.03)	150	SNX13	1.2%		ReMIC(β=0.01); ReMIC(β=0.02); MUFFINN(DNsum)
51	FLI1	2.4%		ReMIC(β=0.01); ReMIC(β=0.02); ReMIC(β=0.03)	151	LGALS3BP	1.2%		HotNet2; ReMIC(β=0.01); ReMIC(β=0.02); MUFFINN(DNsum)
52	LDHA	2.4%		ReMIC(β=0.01); ReMIC(β=0.02); ReMIC(β=0.03)	152	MDM4	1.2%	CGC	ReMIC(β=0.01); ReMIC(β=0.02); ReMIC(β=0.03); MUFFINN(DNmax); MUFFINN(DNsum)
53	TSC2	2.4%		ReMIC(β=0.01); ReMIC(β=0.02); ReMIC(β=0.03)	153	ANXA1	1.2%		HotNet2; ReMIC(β=0.01); ReMIC(β=0.02); ReMIC(β=0.03); MUFFINN(DNmax); MUFFINN(DNsum)
54	TERT	2.4%	Novel	ReMIC(β=0.01); ReMIC(β=0.02); ReMIC(β=0.03)	154	MAPK7	1.2%		ReMIC(β=0.01); MUFFINN(DNsum)

55	LAMP1	2.4%		ReMIC(β=0.01); ReMIC(β=0.02); ReMIC(β=0.03)
56	COL3A1	2.4%		HotNet2; ReMIC(β=0.01); ReMIC(β=0.02); ReMIC(β=0.03)
57	ABCC4	2.4%		HotNet2; ReMIC(β=0.01); ReMIC(β=0.02); ReMIC(β=0.03)
58	EP400	2.4%		ReMIC(β=0.01); ReMIC(β=0.02); ReMIC(β=0.03)
59	ROS1	2.4%	CGC	HotNet2; ReMIC(β=0.01); ReMIC(β=0.02); ReMIC(β=0.03)
60	SIK2	2.4%		ReMIC(β=0.01); ReMIC(β=0.02); ReMIC(β=0.03)
61	AGAP2	2.4%		ReMIC(β=0.01); ReMIC(β=0.02); ReMIC(β=0.03)
62	MSI1	2.4%		ReMIC(β=0.01); ReMIC(β=0.02); ReMIC(β=0.03)
63	KAT6B	2.4%		ReMIC(β=0.01); ReMIC(β=0.02); ReMIC(β=0.03)
64	TRIM3	2.4%		ReMIC(β=0.01); ReMIC(β=0.02); ReMIC(β=0.03)
65	SOX11	2.4%		ReMIC(β=0.01); ReMIC(β=0.02); ReMIC(β=0.03)
66	SLC12A6	2.4%		ReMIC(β=0.01); ReMIC(β=0.02); ReMIC(β=0.03)
67	CTNNB1	1.2%		ReMIC(β=0.01); ReMIC(β=0.02); ReMIC(β=0.03); MUFFINN(DNsum)
68	BCL2	1.2%		ReMIC(β=0.01); ReMIC(β=0.02); ReMIC(β=0.03); MUFFINN(DNmax); MUFFINN(DNsum)
69	USF1	1.2%		ReMIC(β=0.01); ReMIC(β=0.02); ReMIC(β=0.03); MUFFINN(DNsum)
70	GNAS	1.2%		ReMIC(β=0.01); MUFFINN(DNsum)
71	STK36	1.2%		HotNet2; ReMIC(β=0.01); ReMIC(β=0.02); ReMIC(β=0.03); MUFFINN(DNmax); MUFFINN(DNsum)
72	CBL	1.2%		ReMIC(β=0.01); ReMIC(β=0.02); ReMIC(β=0.03)
73	BAX	1.2%		ReMIC(β=0.01); ReMIC(β=0.02); MUFFINN(DNmax); MUFFINN(DNsum)
74	ILK	1.2%		ReMIC(β=0.01); ReMIC(β=0.02); ReMIC(β=0.03); MUFFINN(DNsum)
75	ERBB3	1.2%		ReMIC(β=0.01); ReMIC(β=0.02); ReMIC(β=0.03); MUFFINN(DNmax); MUFFINN(DNsum)
76	SMAD4	1.2%		ReMIC(β=0.01); ReMIC(β=0.02); ReMIC(β=0.03); MUFFINN(DNmax); MUFFINN(DNsum)
77	STAT1	1.2%		ReMIC(β=0.01); ReMIC(β=0.02); ReMIC(β=0.03); MUFFINN(DNsum)
78	LYN	1.2%		ReMIC(β=0.01); ReMIC(β=0.02); ReMIC(β=0.03); MUFFINN(DNsum)
79	PRKCD	1.2%		ReMIC(β=0.01); ReMIC(β=0.02); ReMIC(β=0.03); MUFFINN(DNsum)
80	WEE1	1.2%		ReMIC(β=0.01); ReMIC(β=0.03); MUFFINN(DNsum)
81	PRKCA	1.2%		ReMIC(β=0.01); ReMIC(β=0.02); ReMIC(β=0.03); MUFFINN(DNmax); MUFFINN(DNsum)
82	FGFR2	1.2%		HotNet2; ReMIC(β=0.01); ReMIC(β=0.02); MUFFINN(DNsum)
155	NDUFA10	1.2%		ReMIC(β=0.01); MUFFINN(DNsum)
156	MEOX2	1.2%		MUFFINN(DNmax); MUFFINN(DNsum)
157	SLC25A13	1.2%		ReMIC(β=0.01); MUFFINN(DNmax); MUFFINN(DNsum)
158	KIF3B	1.2%		MUFFINN(DNsum)
159	FLT4	1.2%		ReMIC(β=0.01); ReMIC(β=0.02); ReMIC(β=0.03); MUFFINN(DNsum)
160	UNG	1.2%		ReMIC(β=0.01); MUFFINN(DNsum)
161	NELL2	1.2%		ReMIC(β=0.01); MUFFINN(DNsum)
162	APBB1IP	1.2%		MUFFINN(DNmax); MUFFINN(DNsum)
163	GYPC	1.2%		ReMIC(β=0.01); MUFFINN(DNsum)
164	WNT2	1.2%		MUFFINN(DNmax); MUFFINN(DNsum)
165	SYP	1.2%		ReMIC(β=0.01); MUFFINN(DNsum)
166	ZNF384	1.2%		ReMIC(β=0.01)
167	KLF4	1.2%		HotNet2; ReMIC(β=0.01); ReMIC(β=0.02); ReMIC(β=0.03)
168	CCNB1	1.2%		ReMIC(β=0.01); ReMIC(β=0.02); ReMIC(β=0.03); MUFFINN(DNsum)
169	KTN1	1.2%		ReMIC(β=0.01); MUFFINN(DNsum)
170	KPNA2	1.2%		ReMIC(β=0.01); ReMIC(β=0.02); ReMIC(β=0.03); MUFFINN(DNsum)
171	ELAVL2	1.2%		ReMIC(β=0.01); MUFFINN(DNsum)
172	DTX3	1.2%		MUFFINN(DNsum)
173	CDC123	1.2%		MUFFINN(DNmax); MUFFINN(DNsum)
174	NRAS	1.2%		ReMIC(β=0.01); MUFFINN(DNsum)
175	PPP2R5D	1.2%		HotNet2; ReMIC(β=0.01); ReMIC(β=0.02); MUFFINN(DNsum)
176	FGFR1	1.2%		ReMIC(β=0.01); ReMIC(β=0.02); ReMIC(β=0.03); MUFFINN(DNsum)
177	TNFRSF11B	1.2%		MUFFINN(DNsum)
178	CAST	1.2%		HotNet2; ReMIC(β=0.01); ReMIC(β=0.02); ReMIC(β=0.03); MUFFINN(DNsum)
179	TAOK3	1.2%		ReMIC(β=0.01); MUFFINN(DNsum)
180	GRIA2	1.2%		MUFFINN(DNsum)
181	FOXO4	1.2%		ReMIC(β=0.01); ReMIC(β=0.02); ReMIC(β=0.03); MUFFINN(DNmax); MUFFINN(DNsum)
182	MYCN	1.2%		HotNet2; ReMIC(β=0.01); ReMIC(β=0.02); ReMIC(β=0.03); MUFFINN(DNmax); MUFFINN(DNsum)

83	NOS3	1.2%		ReMIC(β=0.01); ReMIC(β=0.02)	183	PMS2	1.2%		ReMIC(β=0.01); ReMIC(β=0.02); ReMIC(β=0.03); MUFFINN(DNmax); MUFFINN(DNsum)
84	SHH	1.2%		HotNet2; ReMIC(β=0.01); MUFFINN(DNsum)	184	PLAG1	1.2%		ReMIC(β=0.01); ReMIC(β=0.02); ReMIC(β=0.03); MUFFINN(DNsum)
85	ATP6V1E1	1.2%		ReMIC(β=0.01); ReMIC(β=0.02); MUFFINN(DNmax); MUFFINN(DNsum)	185	MYLK2	1.2%		ReMIC(β=0.01); MUFFINN(DNsum)
86	ATR	1.2%		ReMIC(β=0.01); ReMIC(β=0.02); ReMIC(β=0.03); MUFFINN(DNmax); MUFFINN(DNsum)	186	LAX1	1.2%		ReMIC(β=0.01); MUFFINN(DNmax); MUFFINN(DNsum)
87	VAV2	1.2%		ReMIC(β=0.01); ReMIC(β=0.02); ReMIC(β=0.03); MUFFINN(DNmax); MUFFINN(DNsum)	187	GLI1	1.2%		HotNet2; ReMIC(β=0.01); MUFFINN(DNsum)
88	TOP1	1.2%		ReMIC(β=0.01); ReMIC(β=0.02); ReMIC(β=0.03); MUFFINN(DNsum)	188	AVIL	1.2%		ReMIC(β=0.01); ReMIC(β=0.02); MUFFINN(DNmax); MUFFINN(DNsum)
89	CSNK1E	1.2%		ReMIC(β=0.01); ReMIC(β=0.02); ReMIC(β=0.03); MUFFINN(DNsum)	189	NTRK3	1.2%		ReMIC(β=0.01); MUFFINN(DNsum)
90	CYLD	1.2%		ReMIC(β=0.01); ReMIC(β=0.02); MUFFINN(DNmax); MUFFINN(DNsum)	190	JAG1	1.2%		HotNet2; ReMIC(β=0.01); ReMIC(β=0.02); MUFFINN(DNsum)
91	STAT3	1.2%		ReMIC(β=0.01); ReMIC(β=0.02); ReMIC(β=0.03); MUFFINN(DNsum)	191	BAMBI	1.2%		ReMIC(β=0.01); MUFFINN(DNmax); MUFFINN(DNsum)
92	SPARC	1.2%		HotNet2; ReMIC(β=0.01); ReMIC(β=0.02); ReMIC(β=0.03); MUFFINN(DNsum)	192	CHL1	1.2%		ReMIC(β=0.01); ReMIC(β=0.02); MUFFINN(DNsum)
93	RPN1	1.2%		MUFFINN(DNsum)	193	EPG5	1.2%		ReMIC(β=0.01); MUFFINN(DNsum)
94	ATM	1.2%		ReMIC(β=0.01); ReMIC(β=0.02); ReMIC(β=0.03)	194	CDKL5	1.2%		ReMIC(β=0.01); MUFFINN(DNsum)
95	PHLPP1	1.2%		MUFFINN(DNsum)	195	CYP3A4	1.2%		ReMIC(β=0.01); MUFFINN(DNsum)
96	TBK1	1.2%		ReMIC(β=0.01); ReMIC(β=0.02); ReMIC(β=0.03); MUFFINN(DNmax); MUFFINN(DNsum)	196	SLC2A2	1.2%		ReMIC(β=0.01); ReMIC(β=0.02); MUFFINN(DNsum)
97	SOCS1	1.2%		ReMIC(β=0.01); ReMIC(β=0.02); ReMIC(β=0.03); MUFFINN(DNmax); MUFFINN(DNsum)	197	COL6A2	1.2%		ReMIC(β=0.01); MUFFINN(DNmax); MUFFINN(DNsum)
98	UPF2	1.2%		MUFFINN(DNsum)	198	ARHGAP35	1.2%	IntOGen	ReMIC(β=0.01); MUFFINN(DNmax); MUFFINN(DNsum)
99	INSR	1.2%		ReMIC(β=0.01); ReMIC(β=0.02); ReMIC(β=0.03); MUFFINN(DNsum)	199	DMBT1	1.2%		ReMIC(β=0.01); MUFFINN(DNsum)
100	AIFM1	1.2%		ReMIC(β=0.01); ReMIC(β=0.02); MUFFINN(DNmax); MUFFINN(DNsum)	200	FLT1	1.2%		HotNet2; ReMIC(β=0.01); ReMIC(β=0.02); ReMIC(β=0.03); MUFFINN(DNmax); MUFFINN(DNsum)

Table S4. The full list of the top 200 genes detected by our model on COADREAD dataset. The table demonstrates the ranks of the genes, gene symbols, mutation rates, the included benchmarking gene lists [1, 2] of the genes and the other methods [5, 6, 7] by which the genes are also detected.

Rank	Symbol	MutRate	Benchmarking	OtherMethod	Rank	Symbol	MutRate	Benchmarking	OtherMethod
1	APC	77.3%	CGC; IntOGen	HotNet2; ReMIC(β=0.01); ReMIC(β=0.02); ReMIC(β=0.03)	101	DNM1	7.2%		ReMIC(β=0.01); ReMIC(β=0.02); ReMIC(β=0.03)
2	TP53	54.6%	CGC; IntOGen		102	TNR	8.2%		
3	KRAS	42.0%	CGC; IntOGen	HotNet2	103	COL12A1	10.6%		ReMIC(β=0.01); ReMIC(β=0.02); ReMIC(β=0.03)
4	TTN	40.1%			104	LRP6	7.2%		
5	SYNE1	28.5%			105	IGF2R	8.7%		HotNet2
6	MUC16	20.3%			106	MSH6	8.7%	CGC	
7	LRP1B	21.7%			107	PCLO	8.7%		
8	PIK3CA	18.4%	CGC; IntOGen		108	VWF	8.7%		
9	LRP2	17.4%			109	SLX4	6.8%		
10	FBXW7	16.9%	CGC; IntOGen	ReMIC(β=0.01); ReMIC(β=0.02)	110	TRRAP	7.7%		
11	DMD	16.9%		HotNet2; ReMIC(β=0.01); ReMIC(β=0.02); ReMIC(β=0.03)	111	EVC2	11.1%		ReMIC(β=0.01); ReMIC(β=0.02); ReMIC(β=0.03)
12	FLG	16.9%		ReMIC(β=0.01); ReMIC(β=0.02)	112	PRKDC	7.7%		
13	FAT4	20.3%		ReMIC(β=0.01); ReMIC(β=0.02); ReMIC(β=0.03)	113	TAF1L	9.2%		

14	CUBN	14.5%		HotNet2; ReMIC(β=0.01); ReMIC(β=0.02); ReMIC(β=0.03)
15	DNAH5	18.4%		ReMIC(β=0.01); ReMIC(β=0.02); ReMIC(β=0.03)
16	ANK2	14.0%		HotNet2; ReMIC(β=0.01); ReMIC(β=0.02); ReMIC(β=0.03)
17	CSMD1	15.0%		ReMIC(β=0.01); ReMIC(β=0.02); ReMIC(β=0.03)
18	RYR1	13.0%		
19	USH2A	16.9%		
20	LAMA1	13.5%		HotNet2; MUFFINN(DNsum)
21	SMAD4	11.6%	CGC; IntOGen	
22	GRIN2A	12.6%	Novel	HotNet2
23	RYR2	12.6%		
24	TCF7L2	14.0%	CGC; IntOGen	
25	RELN	12.6%		
26	VCAN	11.6%		
27	MACF1	12.1%		
28	NEB	10.6%		
29	APOB	11.6%		ReMIC(β=0.01); ReMIC(β=0.02); ReMIC(β=0.03)
30	COL6A3	15.5%		ReMIC(β=0.01); ReMIC(β=0.02); ReMIC(β=0.03)
31	ATM	10.6%	IntOGen	ReMIC(β=0.01); ReMIC(β=0.02); ReMIC(β=0.03)
32	CSMD2	14.5%		ReMIC(β=0.01); ReMIC(β=0.02); ReMIC(β=0.03)
33	ROBO1	11.1%		
34	ERBB4	10.6%		ReMIC(β=0.01); ReMIC(β=0.02); ReMIC(β=0.03)
35	FN1	9.7%	IntOGen	ReMIC(β=0.01); ReMIC(β=0.02)
36	DOCK2	11.1%		HotNet2; ReMIC(β=0.01); ReMIC(β=0.02); ReMIC(β=0.03)
37	MYH11	11.1%		
38	ANK1	10.6%		HotNet2; ReMIC(β=0.01); ReMIC(β=0.02); ReMIC(β=0.03); MUFFINN(DNmax)
39	PKHD1	12.1%		
40	ARID1A	11.1%	IntOGen	ReMIC(β=0.01); ReMIC(β=0.02); ReMIC(β=0.03)
41	TNC	11.1%		
42	COL5A1	10.6%		HotNet2; ReMIC(β=0.01); ReMIC(β=0.02); ReMIC(β=0.03)
43	AKAP9	9.7%	IntOGen	ReMIC(β=0.01); ReMIC(β=0.02); ReMIC(β=0.03); MUFFINN(DNmax)
44	MAP1B	10.6%		
45	LRP1	9.2%		
46	NRXN1	10.6%		
47	CREBBP	9.2%	IntOGen	ReMIC(β=0.01); ReMIC(β=0.02); ReMIC(β=0.03)
48	SDK1	14.5%		
49	RYR3	11.6%		
50	LIFR	10.6%		
51	SACS	12.1%		
52	FBN1	9.7%		ReMIC(β=0.01); ReMIC(β=0.02); ReMIC(β=0.03)
53	MDN1	11.6%		
54	DYNC1H1	9.7%		ReMIC(β=0.01); ReMIC(β=0.02); ReMIC(β=0.03)
55	DAPK1	9.2%		ReMIC(β=0.01); ReMIC(β=0.02); ReMIC(β=0.03)
56	MAP2	10.6%		
114	ACVR2A	9.7%		HotNet2; ReMIC(β=0.01); ReMIC(β=0.02); ReMIC(β=0.03); MUFFINN(DNmax)
115	ABCA1	7.2%		ReMIC(β=0.01); ReMIC(β=0.02); ReMIC(β=0.03); MUFFINN(DNmax)
116	GRIA1	8.2%		ReMIC(β=0.01)
117	DNMT1	8.2%		ReMIC(β=0.01); ReMIC(β=0.02); ReMIC(β=0.03)
118	SCN1A	9.7%		
119	BAI3	9.7%		ReMIC(β=0.01); ReMIC(β=0.02); ReMIC(β=0.03)
120	C3	6.8%		ReMIC(β=0.01); ReMIC(β=0.02); ReMIC(β=0.03)
121	ABCA12	11.1%		ReMIC(β=0.01); ReMIC(β=0.02); ReMIC(β=0.03)
122	REV3L	8.7%		
123	SPTB	8.7%		
124	HUWE1	8.2%		
125	SMAD2	6.8%	CGC; IntOGen	
126	SRCAP	8.2%		
127	PTPRM	9.7%		
128	NFASC	8.2%		
129	EDNRB	7.7%		ReMIC(β=0.01); ReMIC(β=0.02); ReMIC(β=0.03)
130	CAD	7.2%	IntOGen	ReMIC(β=0.01); ReMIC(β=0.02); ReMIC(β=0.03)
131	MYO9A	8.2%		
132	RP1	7.7%		
133	CACHD1	7.7%		ReMIC(β=0.01); ReMIC(β=0.02); ReMIC(β=0.03)
134	CDH2	7.2%		ReMIC(β=0.01); ReMIC(β=0.02); ReMIC(β=0.03)
135	CDH10	8.2%		HotNet2; ReMIC(β=0.01); ReMIC(β=0.02); ReMIC(β=0.03)
136	LAMA3	7.2%		HotNet2
137	NOTCH2	7.2%		
138	SRRM2	6.8%		
139	ZFHX3	9.2%		
140	POLE	7.2%	Novel	
141	COL4A5	7.7%		HotNet2; ReMIC(β=0.01); ReMIC(β=0.02); ReMIC(β=0.03)
142	CHD6	8.2%		ReMIC(β=0.01); ReMIC(β=0.02); ReMIC(β=0.03)
143	CDH18	7.7%		HotNet2; ReMIC(β=0.01); ReMIC(β=0.02); ReMIC(β=0.03)
144	CDH12	7.2%		HotNet2; ReMIC(β=0.01); ReMIC(β=0.02); ReMIC(β=0.03)
145	MCC	6.3%		
146	DNAH3	10.6%		ReMIC(β=0.01); ReMIC(β=0.02); ReMIC(β=0.03)
147	MAP3K4	7.7%	IntOGen	
148	SCN5A	7.7%		
149	SORCS1	8.7%		
150	STAB2	7.7%		
151	HERC2	7.7%		
152	PCDHB5	7.2%		
153	TNS1	8.7%		
154	ATP2B2	8.2%		ReMIC(β=0.01); ReMIC(β=0.02); ReMIC(β=0.03)
155	SCN2A	7.7%		
156	DMXL2	7.7%		HotNet2; ReMIC(β=0.01); ReMIC(β=0.02); ReMIC(β=0.03)

57	CHD4	9.2%	IntOGen	ReMIC(β=0.01); ReMIC(β=0.02); ReMIC(β=0.03)
58	ATR	8.7%		ReMIC(β=0.01); ReMIC(β=0.02); ReMIC(β=0.03)
59	EPHA5	9.7%		ReMIC(β=0.01); ReMIC(β=0.02); ReMIC(β=0.03)
60	PLXNA4	11.6%		
61	COL1A2	9.2%		ReMIC(β=0.01); ReMIC(β=0.02); ReMIC(β=0.03)
62	AFF2	11.6%		ReMIC(β=0.01); ReMIC(β=0.02); ReMIC(β=0.03); MUFFINN(DNmax)
63	ADCY2	9.7%		HotNet2; ReMIC(β=0.01); ReMIC(β=0.02); ReMIC(β=0.03); MUFFINN(DNmax); MUFFINN(DNsum)
64	EP300	7.2%	CGC	ReMIC(β=0.01)
65	DST	8.7%		ReMIC(β=0.01); ReMIC(β=0.02); ReMIC(β=0.03)
66	NRXN3	10.1%		
67	EPHA3	10.1%		ReMIC(β=0.01); ReMIC(β=0.02); ReMIC(β=0.03); MUFFINN(DNsum)
68	UTRN	8.7%		MUFFINN(DNsum)
69	FBN2	9.7%		ReMIC(β=0.01); ReMIC(β=0.02)
70	BRAF	9.7%	CGC; IntOGen	ReMIC(β=0.01); ReMIC(β=0.02); ReMIC(β=0.03)
71	TRPC4	9.2%		
72	ZC3H13	9.7%		
73	LYST	8.7%		
74	PDZD2	9.7%		
75	DYSF	9.2%		HotNet2; ReMIC(β=0.01); ReMIC(β=0.02); ReMIC(β=0.03)
76	ALK	8.2%		ReMIC(β=0.01); ReMIC(β=0.02); ReMIC(β=0.03); MUFFINN(DNmax)
77	ACACA	8.2%		ReMIC(β=0.01); ReMIC(β=0.02)
78	LAMA2	10.1%		HotNet2
79	TG	9.7%		
80	CDC27	8.2%		ReMIC(β=0.01); ReMIC(β=0.02); ReMIC(β=0.03)
81	WDFY3	10.1%		
82	ANK3	8.7%		ReMIC(β=0.01); ReMIC(β=0.02); ReMIC(β=0.03)
83	KALRN	9.2%		
84	DCC	8.2%		ReMIC(β=0.01); ReMIC(β=0.02); ReMIC(β=0.03)
85	TGFBR2	9.2%	CGC; IntOGen	
86	MTOR	8.7%		MUFFINN(DNsum)
87	KCNQ3	8.2%		HotNet2
88	NID1	8.2%		
89	NLRP4	8.7%		
90	CTNND2	9.2%		HotNet2; ReMIC(β=0.01); ReMIC(β=0.02); ReMIC(β=0.03)
91	NRAS	9.2%	IntOGen	
92	MYO16	10.1%		
93	GRIK3	9.2%		ReMIC(β=0.01)
94	FCGBP	10.6%		ReMIC(β=0.01); ReMIC(β=0.02)
95	MKI67	9.2%		
96	TIAM1	8.2%		
97	COL11A1	8.7%		HotNet2; ReMIC(β=0.01); ReMIC(β=0.02); ReMIC(β=0.03)
98	GLI3	7.7%		ReMIC(β=0.01)
99	CDH9	8.2%		HotNet2; ReMIC(β=0.01); ReMIC(β=0.02); ReMIC(β=0.03)
157	BSN	7.2%		HotNet2; ReMIC(β=0.01); ReMIC(β=0.02); ReMIC(β=0.03)
158	RIMS2	7.7%		MUFFINN(DNsum)
159	TRIP12	7.7%		
160	FLT1	7.2%		ReMIC(β=0.01); ReMIC(β=0.02)
161	NAV2	7.7%		
162	OBSCN	8.2%		
163	PTPRT	8.7%		
164	UNC79	8.7%		
165	RIF1	6.3%		
166	ASPM	9.2%	IntOGen	ReMIC(β=0.01); ReMIC(β=0.02); ReMIC(β=0.03)
167	MAGEC1	6.8%		HotNet2
168	DSCAML1	7.7%		HotNet2; ReMIC(β=0.01); ReMIC(β=0.02); ReMIC(β=0.03)
169	NIPBL	7.7%		
170	GOLGB1	6.8%		ReMIC(β=0.01)
171	DLC1	8.2%		ReMIC(β=0.01); ReMIC(β=0.02); ReMIC(β=0.03)
172	MYO3A	7.7%		
173	PDZRN4	7.2%		
174	ITPR3	8.2%		HotNet2
175	VPS13D	7.7%		
176	PREX1	7.7%		
177	LRRK2	8.7%		
178	HDAC9	6.8%		
179	COL1A1	7.2%		ReMIC(β=0.01); ReMIC(β=0.02); ReMIC(β=0.03)
180	SPEN	7.2%		
181	NAV3	7.2%		
182	MASP1	7.2%		HotNet2
183	PCDH17	9.7%		
184	F5	6.3%		HotNet2; ReMIC(β=0.01); ReMIC(β=0.02); ReMIC(β=0.03)
185	ARID2	7.2%		ReMIC(β=0.01); ReMIC(β=0.02); ReMIC(β=0.03)
186	TSHZ3	9.2%		
187	CNTN1	7.2%		ReMIC(β=0.01); ReMIC(β=0.02); ReMIC(β=0.03)
188	NCKAP5	7.2%		
189	CENPF	8.2%		ReMIC(β=0.01); ReMIC(β=0.02); ReMIC(β=0.03)
190	THBS2	8.2%		
191	MED12	6.8%		MUFFINN(DNsum)
192	GUCY1A3	7.2%		
193	BRCA2	6.3%		ReMIC(β=0.01); ReMIC(β=0.02); ReMIC(β=0.03)
194	PIK3CG	6.8%		
195	ITPR1	6.3%		
196	EGFR	5.8%	IntOGen	ReMIC(β=0.01); ReMIC(β=0.02); ReMIC(β=0.03)
197	SETD2	7.2%		
198	USP44	6.3%		
199	SEC63	7.7%		

100	CACNA1E	10.1%		HotNet2; ReMIC(β=0.01); ReMIC(β=0.02); ReMIC(β=0.03)	200	PI4KA	7.2%		

Table S5. The full list of the top 200 genes detected by our model on BRCA dataset. The table demonstrates the ranks of the genes, gene symbols, mutation rates, the included benchmarking gene lists [1, 2] of the genes and the other methods [5, 6, 7] by which the genes are also detected.

Rank	Symbol	MutRate	Benchmarking	OtherMethod	Rank	Symbol	MutRate	Benchmarking	OtherMethod
1	TP53	37.6%	CGC; IntOGen		101	WDFY3	2.6%		
2	PIK3CA	35.2%	CGC; IntOGen	ReMIC(β=0.01); ReMIC(β=0.02)	102	MAP1A	2.0%		ReMIC(β=0.01); ReMIC(β=0.02); ReMIC(β=0.03)
3	TTN	17.1%			103	CBLB	2.0%		ReMIC(β=0.01); ReMIC(β=0.02); ReMIC(β=0.03)
4	MUC16	10.9%		HotNet2; ReMIC(β=0.01); ReMIC(β=0.02); ReMIC(β=0.03)	104	SRCAP	2.4%		
5	GATA3	10.9%	CGC; IntOGen	HotNet2; ReMIC(β=0.01); ReMIC(β=0.02); ReMIC(β=0.03)	105	SMARCA4	1.6%	IntOGen	
6	MAP3K1	8.0%	CGC; IntOGen	ReMIC(β=0.01); ReMIC(β=0.02); ReMIC(β=0.03)	106	CACNA1H	2.2%		ReMIC(β=0.01); ReMIC(β=0.02); ReMIC(β=0.03)
7	RYR2	6.6%		ReMIC(β=0.01)	107	CSPG4	2.2%		ReMIC(β=0.01); ReMIC(β=0.02); ReMIC(β=0.03)
8	CDH1	6.8%	CGC; IntOGen	ReMIC(β=0.01); ReMIC(β=0.02); ReMIC(β=0.03)	108	SCN2A	2.0%		ReMIC(β=0.01)
9	OBSCN	6.0%		HotNet2; ReMIC(β=0.01); ReMIC(β=0.02)	109	HRNR	2.2%		ReMIC(β=0.01); ReMIC(β=0.02); ReMIC(β=0.03)
10	MUC4	5.2%		HotNet2; ReMIC(β=0.01); ReMIC(β=0.02); ReMIC(β=0.03)	110	CSMD2	2.4%		ReMIC(β=0.01); ReMIC(β=0.02); ReMIC(β=0.03)
11	USH2A	6.4%			111	HERC2	2.0%		ReMIC(β=0.01); ReMIC(β=0.02); ReMIC(β=0.03)
12	CSMD1	5.6%		ReMIC(β=0.01); ReMIC(β=0.02); ReMIC(β=0.03)	112	SDK1	2.6%		ReMIC(β=0.01)
13	FLG	5.6%		ReMIC(β=0.01); ReMIC(β=0.02); ReMIC(β=0.03)	113	TBL1XR1	2.0%	IntOGen	
14	BRCA2	4.8%	CGC; IntOGen	ReMIC(β=0.01); ReMIC(β=0.02); ReMIC(β=0.03)	114	MGAM	2.4%		ReMIC(β=0.01); ReMIC(β=0.02); ReMIC(β=0.03)
15	BRCA1	3.8%	IntOGen	ReMIC(β=0.01); ReMIC(β=0.02); ReMIC(β=0.03)	115	NOTCH2	1.8%	IntOGen	ReMIC(β=0.01)
16	LRP2	4.0%		ReMIC(β=0.01); ReMIC(β=0.02); ReMIC(β=0.03); MUFFINN(DNsum)	116	DNAH1	2.4%		ReMIC(β=0.01); ReMIC(β=0.02); ReMIC(β=0.03)
17	ATM	4.0%	IntOGen	ReMIC(β=0.01); ReMIC(β=0.02); ReMIC(β=0.03)	117	MYH7	2.2%		ReMIC(β=0.01); ReMIC(β=0.02); ReMIC(β=0.03)
18	NCOR1	3.6%	CGC; IntOGen	ReMIC(β=0.01); ReMIC(β=0.02)	118	CBFB	1.8%	IntOGen	ReMIC(β=0.02); ReMIC(β=0.03)
19	SYNE1	4.4%			119	RPGR	2.0%	IntOGen	ReMIC(β=0.01)
20	PIK3R1	3.0%	IntOGen	ReMIC(β=0.01); ReMIC(β=0.02)	120	PDE3A	2.2%		ReMIC(β=0.01); ReMIC(β=0.02)
21	RYR3	4.8%		ReMIC(β=0.01)	121	LAMA2	2.0%		HotNet2; ReMIC(β=0.01); ReMIC(β=0.02); ReMIC(β=0.03)
22	SPEN	3.8%			122	PREX2	2.6%		ReMIC(β=0.01); ReMIC(β=0.02)
23	SSPO	3.8%			123	EVPL	1.8%		HotNet2; ReMIC(β=0.01); ReMIC(β=0.02); ReMIC(β=0.03); MUFFINN(DNsum)
24	MAP2K4	4.2%	CGC; IntOGen	ReMIC(β=0.01); ReMIC(β=0.02); ReMIC(β=0.03)	124	TEP1	2.0%		
25	RYR1	3.4%		ReMIC(β=0.01)	125	IARS	1.6%		ReMIC(β=0.01)
26	NEB	3.8%		HotNet2; ReMIC(β=0.01); ReMIC(β=0.02)	126	MUC12	2.2%		ReMIC(β=0.01); ReMIC(β=0.02); ReMIC(β=0.03)
27	APOB	3.8%		HotNet2; ReMIC(β=0.01); ReMIC(β=0.02); ReMIC(β=0.03)	127	VCAN	1.8%		
28	RUNX1	3.8%	IntOGen	ReMIC(β=0.01)	128	ABCB1	1.8%		ReMIC(β=0.01); ReMIC(β=0.02); ReMIC(β=0.03); MUFFINN(DNmax)
29	PTEN	3.6%	IntOGen	ReMIC(β=0.01)	129	MED23	1.8%		ReMIC(β=0.01); ReMIC(β=0.02); ReMIC(β=0.03)
30	SPI1	3.2%			130	MAST1	1.8%		ReMIC(β=0.01); ReMIC(β=0.03)
31	SYNE2	3.8%			131	HUWE1	1.8%		ReMIC(β=0.02); ReMIC(β=0.03)
32	SPTA1	3.6%			132	HTT	1.6%		ReMIC(β=0.01); ReMIC(β=0.02); ReMIC(β=0.03)

33	UBR4	3.6%			133	MYH14	2.0%	IntOGen	HotNet2; ReMIC(β=0.01); ReMIC(β=0.02); ReMIC(β=0.03)
34	MTOR	2.6%	IntOGen	ReMIC(β=0.01); ReMIC(β=0.02); ReMIC(β=0.03)	134	SVIL	1.6%		
35	DMD	3.2%		HotNet2; ReMIC(β=0.01); ReMIC(β=0.02); ReMIC(β=0.03)	135	PTPRB	1.8%		ReMIC(β=0.01)
36	AKT1	2.6%	CGC; IntOGen	ReMIC(β=0.01); ReMIC(β=0.02); ReMIC(β=0.03)	136	GRIN2A	2.0%		ReMIC(β=0.01); ReMIC(β=0.02); ReMIC(β=0.03)
37	FBN1	2.6%		ReMIC(β=0.01); ReMIC(β=0.02); ReMIC(β=0.03)	137	COL14A1	2.2%		ReMIC(β=0.01); ReMIC(β=0.02); ReMIC(β=0.03)
38	ANK1	2.8%		HotNet2; ReMIC(β=0.01); ReMIC(β=0.02); ReMIC(β=0.03)	138	ASPM	2.0%	IntOGen	ReMIC(β=0.01); ReMIC(β=0.02); ReMIC(β=0.03)
39	TG	3.0%			139	FANCA	1.6%		ReMIC(β=0.02); ReMIC(β=0.03)
40	VWF	2.6%			140	KIF4A	1.8%		HotNet2; ReMIC(β=0.01); ReMIC(β=0.02); ReMIC(β=0.03)
41	RELN	3.4%		ReMIC(β=0.01)	141	TPR	1.8%		
42	FLNC	2.8%		ReMIC(β=0.01); ReMIC(β=0.02); ReMIC(β=0.03)	142	MYH6	1.8%		HotNet2; ReMIC(β=0.01); ReMIC(β=0.03)
43	MUC2	3.6%		ReMIC(β=0.01); ReMIC(β=0.02); ReMIC(β=0.03)	143	GRM3	1.8%		ReMIC(β=0.01); ReMIC(β=0.03)
44	LAMA5	2.6%		ReMIC(β=0.01); ReMIC(β=0.02); ReMIC(β=0.03)	144	PIWIL1	1.8%		ReMIC(β=0.01); ReMIC(β=0.02)
45	PLEC	3.0%		ReMIC(β=0.01); ReMIC(β=0.02)	145	PREX1	1.8%		ReMIC(β=0.02)
46	CTCF	3.0%	CGC; IntOGen	ReMIC(β=0.01); ReMIC(β=0.02); ReMIC(β=0.03)	146	TAF1	1.6%	IntOGen	
47	LRP1	2.4%		ReMIC(β=0.01); ReMIC(β=0.02); ReMIC(β=0.03)	147	COL12A1	2.2%		ReMIC(β=0.01); ReMIC(β=0.02); ReMIC(β=0.03)
48	MDN1	3.0%		ReMIC(β=0.01); ReMIC(β=0.02); ReMIC(β=0.03)	148	MADD	2.0%		HotNet2; ReMIC(β=0.01); ReMIC(β=0.02); ReMIC(β=0.03)
49	MLLT4	2.4%	IntOGen	ReMIC(β=0.01); ReMIC(β=0.02); ReMIC(β=0.03)	149	FLNB	1.8%		ReMIC(β=0.01); ReMIC(β=0.02); ReMIC(β=0.03)
50	DST	2.8%		ReMIC(β=0.01); ReMIC(β=0.02); ReMIC(β=0.03)	150	NLRC4	1.8%		HotNet2; ReMIC(β=0.01); ReMIC(β=0.02)
51	NF1	2.8%	IntOGen	ReMIC(β=0.01); ReMIC(β=0.02)	151	ERBB2	1.6%	CGC; IntOGen	ReMIC(β=0.01); ReMIC(β=0.02); ReMIC(β=0.03)
52	TLN1	2.0%			152	CENPE	2.0%		ReMIC(β=0.01); ReMIC(β=0.02); ReMIC(β=0.03)
53	MYO10	2.6%		ReMIC(β=0.01); ReMIC(β=0.02)	153	PLXNA2	1.6%		ReMIC(β=0.01); ReMIC(β=0.02)
54	CACNA1B	2.6%		ReMIC(β=0.01); ReMIC(β=0.02); ReMIC(β=0.03)	154	SETDB1	1.4%	IntOGen	
55	XIRP2	3.0%			155	ASMTL	1.6%		
56	MACF1	2.2%	IntOGen	ReMIC(β=0.01); ReMIC(β=0.02); ReMIC(β=0.03)	156	CHD6	1.8%		ReMIC(β=0.01); ReMIC(β=0.02); ReMIC(β=0.03)
57	PRKDC	2.0%		ReMIC(β=0.01); ReMIC(β=0.02)	157	MYCBP2	1.8%		ReMIC(β=0.01); ReMIC(β=0.02)
58	LAMA1	2.4%		ReMIC(β=0.01); ReMIC(β=0.02); ReMIC(β=0.03)	158	MYO7A	1.8%		HotNet2; ReMIC(β=0.02)
59	SVEP1	2.8%	IntOGen		159	SCN10A	1.8%		
60	HSPG2	2.2%		ReMIC(β=0.01); ReMIC(β=0.02); ReMIC(β=0.03)	160	HGFAC	1.6%		ReMIC(β=0.03)
61	CACNA1E	2.8%		HotNet2; ReMIC(β=0.01); ReMIC(β=0.02); ReMIC(β=0.03)	161	MAP2	1.8%		ReMIC(β=0.01); ReMIC(β=0.02); ReMIC(β=0.03)
62	PKHD1	2.6%		ReMIC(β=0.01); ReMIC(β=0.02)	162	UTP20	1.6%		
63	AKAP9	2.2%	IntOGen	ReMIC(β=0.03); MUFFINN(DNmax)	163	NEDD9	1.4%		ReMIC(β=0.02)
64	RB1	1.8%	CGC; IntOGen	ReMIC(β=0.01)	164	MYH9	1.6%	IntOGen	ReMIC(β=0.01); ReMIC(β=0.02); ReMIC(β=0.03)
65	DNAH5	2.6%		ReMIC(β=0.01); ReMIC(β=0.02); ReMIC(β=0.03)	165	CR1	1.8%		ReMIC(β=0.01); ReMIC(β=0.02); ReMIC(β=0.03)
66	F5	2.2%		HotNet2; ReMIC(β=0.01); ReMIC(β=0.02); ReMIC(β=0.03)	166	PKD1	2.0%		ReMIC(β=0.01); ReMIC(β=0.02)
67	BZRAP1	2.2%		ReMIC(β=0.01); ReMIC(β=0.02); ReMIC(β=0.03)	167	CABIN1	1.6%		
68	CHD4	2.2%	IntOGen	ReMIC(β=0.01); ReMIC(β=0.02); ReMIC(β=0.03); MUFFINN(DNsum)	168	DYNC2H1	1.8%		ReMIC(β=0.01); ReMIC(β=0.02); ReMIC(β=0.03)
69	CAPN1	2.2%		ReMIC(β=0.01); ReMIC(β=0.02); ReMIC(β=0.03)	169	MYB	1.6%	IntOGen	ReMIC(β=0.01); ReMIC(β=0.02); ReMIC(β=0.03)
70	ACTN2	1.8%		ReMIC(β=0.01); ReMIC(β=0.02); ReMIC(β=0.03); MUFFINN(DNmax)	170	GOLGB1	1.8%		ReMIC(β=0.01); ReMIC(β=0.03)

71	CACNA1C	2.4%		HotNet2; ReMIC(β=0.01); ReMIC(β=0.02); ReMIC(β=0.03)	171	PLXNA4	2.4%		ReMIC(β=0.01); ReMIC(β=0.02)
72	GRIN2B	2.0%		ReMIC(β=0.01); ReMIC(β=0.02); ReMIC(β=0.03)	172	CNTN1	1.6%		HotNet2; ReMIC(β=0.01); ReMIC(β=0.02); ReMIC(β=0.03)
73	ZFHX3	2.8%			173	DSCAML1	1.8%		HotNet2; ReMIC(β=0.01); ReMIC(β=0.02); ReMIC(β=0.03)
74	UBC	1.4%			174	GRIK2	1.6%		ReMIC(β=0.02)
75	HECW1	2.6%		ReMIC(β=0.01); ReMIC(β=0.02); ReMIC(β=0.03)	175	CACNA1A	2.0%		ReMIC(β=0.01); ReMIC(β=0.02); ReMIC(β=0.03); MUFFINN(DNsum)
76	AFF2	2.8%		ReMIC(β=0.01); ReMIC(β=0.02); ReMIC(β=0.03); MUFFINN(DNmax)	176	ARID1B	2.0%	CGC	ReMIC(β=0.01); ReMIC(β=0.02); ReMIC(β=0.03)
77	ERBB3	1.8%		ReMIC(β=0.01); ReMIC(β=0.02); ReMIC(β=0.03)	177	KIF13A	1.6%		
78	DNAH3	3.0%		ReMIC(β=0.01); ReMIC(β=0.02); ReMIC(β=0.03)	178	TCHH	1.8%		
79	ITPR1	2.0%		ReMIC(β=0.01); ReMIC(β=0.02); ReMIC(β=0.03)	179	BRIP1	2.0%		ReMIC(β=0.01); ReMIC(β=0.02); ReMIC(β=0.03)
80	MED12	2.2%		ReMIC(β=0.01); ReMIC(β=0.02); ReMIC(β=0.03); MUFFINN(DNsum)	180	RLF	1.8%		ReMIC(β=0.01)
81	TBX3	2.8%	CGC; IntOGen		181	KIAA0430	1.6%		HotNet2; ReMIC(β=0.01); ReMIC(β=0.03)
82	SF3B1	2.0%	IntOGen	ReMIC(β=0.01)	182	LRBA	2.2%		ReMIC(β=0.01); ReMIC(β=0.02); ReMIC(β=0.03)
83	DCC	2.0%		ReMIC(β=0.01); ReMIC(β=0.02); ReMIC(β=0.03)	183	NRXN3	1.8%		HotNet2; ReMIC(β=0.01); ReMIC(β=0.02)
84	CROCC	2.4%		ReMIC(β=0.01); ReMIC(β=0.02); ReMIC(β=0.03)	184	TLR4	1.4%		
85	CACNA1G	2.8%		ReMIC(β=0.01); ReMIC(β=0.02); ReMIC(β=0.03)	185	ERBB4	1.6%		ReMIC(β=0.01); ReMIC(β=0.02); ReMIC(β=0.03)
86	WWP2	2.0%			186	PDCD11	1.4%		
87	MYLK	2.0%		HotNet2; ReMIC(β=0.01); ReMIC(β=0.02); ReMIC(β=0.03)	187	NCOA3	1.4%		ReMIC(β=0.02)
88	ZFPM2	2.2%			188	GCN1L1	1.4%		
89	PCNT	2.2%		ReMIC(β=0.01); ReMIC(β=0.02)	189	ANK2	1.4%		ReMIC(β=0.01); ReMIC(β=0.03)
90	ARID1A	2.2%	CGC; IntOGen	ReMIC(β=0.01); ReMIC(β=0.02); ReMIC(β=0.03)	190	HECW2	1.8%		ReMIC(β=0.01); ReMIC(β=0.02); ReMIC(β=0.03)
91	ATN1	1.8%		ReMIC(β=0.01); ReMIC(β=0.02); ReMIC(β=0.03)	191	GRIA1	1.6%		ReMIC(β=0.01); ReMIC(β=0.03)
92	UTRN	2.2%			192	IKBKE	1.2%		
93	UBR5	2.0%			193	CUL7	1.6%		ReMIC(β=0.01); ReMIC(β=0.02)
94	CIT	2.2%		ReMIC(β=0.01); ReMIC(β=0.02); ReMIC(β=0.03)	194	AKAP4	1.6%		ReMIC(β=0.01); ReMIC(β=0.02)
95	EGFR	1.4%	IntOGen	ReMIC(β=0.01); ReMIC(β=0.02); ReMIC(β=0.03)	195	NUP160	1.6%		HotNet2; ReMIC(β=0.01); ReMIC(β=0.02)
96	CFH	2.0%		HotNet2; ReMIC(β=0.01); ReMIC(β=0.02); ReMIC(β=0.03)	196	KCNQ3	1.4%		HotNet2
97	SRRM2	1.8%			197	SEC24B	1.4%		
98	ANK3	2.2%	IntOGen	HotNet2; ReMIC(β=0.01); ReMIC(β=0.02); ReMIC(β=0.03)	198	DNAH7	2.0%		ReMIC(β=0.01); ReMIC(β=0.03)
99	PTPRD	2.2%		ReMIC(β=0.01)	199	COL6A3	2.0%		ReMIC(β=0.01); ReMIC(β=0.02); ReMIC(β=0.03)
100	ATRX	2.0%		ReMIC(β=0.01); ReMIC(β=0.02); ReMIC(β=0.03)	200	RIPK1	1.4%		

Table S6. Functional enrichment analysis results for KEGG pathways [8] of the top 200 genes of the proposed method on COADREAD dataset. The pathways are sorted by their enrichment p-values.

Pathway	Count	%	p-value	Pathway	Count	%	p-value
Focal adhesion	20	10.05	2.15e-09	Thyroid cancer	5	2.51	1.51e-03
Pathways in cancer	27	13.57	2.45e-09	Gap junction	7	3.52	4.29e-03
Colorectal cancer	12	6.03	7.18e-09	GnRH signaling pathway	7	3.52	5.05e-03
Pancreatic cancer	10	5.03	1.61e-06	Melanogenesis	7	3.52	7.96e-03
Prostate cancer	11	5.53	2.66e-06	TGF-beta signaling pathway	6	3.02	1.56e-02
Endometrial cancer	9	4.52	2.77e-06	Chemokine signaling pathway	9	4.52	1.61e-02
Non-small cell lung cancer	8	4.02	4.83e-05	Regulation of actin cytoskeleton	9	4.52	3.14e-02
Glioma	8	4.02	1.27e-04	Wnt signaling pathway	7	3.52	3.37e-02
Adherens junction	8	4.02	2.23e-04	Insulin signaling pathway	7	3.52	3.37e-02
Chronic myeloid leukemia	8	4.02	2.44e-04	Cell adhesion molecules (CAMs)	7	3.52	3.80e-02
Calcium signaling pathway	12	6.03	2.79e-04	Neurotrophin signaling pathway	6	3.02	5.89e-02
Acute myeloid leukemia	7	3.52	4.04e-04	Basal cell carcinoma	4	2.01	7.13e-02
Small cell lung cancer	8	4.02	6.77e-04	Axon guidance	6	3.02	7.16e-02
Bladder cancer	6	3.02	6.86e-04	Dorso-ventral axis formation	3	1.51	8.04e-02
ErbB signaling pathway	8	4.02	7.78e-04	mTOR signaling pathway	4	2.01	8.08e-02
Renal cell carcinoma	7	3.52	9.05e-04	VEGF signaling pathway	4	2.01	9.08e-02
Cell cycle	9	4.52	1.42e-03	Phosphatidylinositol signaling system	5	2.51	9.25e-02
Melanoma	7	3.52	1.44e-03	Apoptosis	4	2.01	9.43e-02

Table S7. Functional enrichment analysis results for KEGG pathways [8] of the top 200 genes of the proposed method on BRCA dataset. The pathways are sorted by their enrichment p-values.

Pathway	Count	%	p-value	Pathway	Count	%	p-value
Calcium signaling pathway	16	8.12	3.11e-07	Chronic myeloid leukemia	7	3.55	1.36e-03
Focal adhesion	17	8.63	3.46e-07	Acute myeloid leukemia	6	3.05	2.54e-03
ErbB signaling pathway	10	5.08	1.53e-05	Bladder cancer	5	2.54	5.03e-03
Endometrial cancer	8	4.06	2.51e-05	Ubiquitin mediated proteolysis	8	4.06	8.77e-03
Small cell lung cancer	9	4.57	9.16e-05	Toll-like receptor signaling pathway	7	3.55	9.31e-03
Pathways in cancer	19	9.64	1.07e-04	mTOR signaling pathway	5	2.54	1.69e-02
Pancreatic cancer	8	4.06	1.09e-04	Colorectal cancer	5	2.54	2.11e-02
Glioma	8	4.06	1.09e-04	Tight junction	7	3.55	2.94e-02
Prostate cancer	9	4.57	1.17e-04	Adherens junction	5	2.54	3.27e-02
Melanoma	8	4.06	1.91e-04	GnRH signaling pathway	5	2.54	6.97e-02
Non-small cell lung cancer	7	3.55	3.53e-04	Dorso-ventral axis formation	3	1.52	7.70e-02
MAPK signaling pathway	14	7.11	3.79e-04	Thyroid cancer	3	1.52	8.71e-02
Apoptosis	7	3.55	6.15e-04				

References

[1] P. A. Futreal, L. Coin, M. Marshall, T. Down, T. Hubbard, R. Wooster, N. Rahman, M. R. Stratton, A census of human cancer genes, Nature Reviews Cancer 4 (3) (2004) 177–183.

[2] A. Gonzalez-Perez, C. Perez-Llamas, J. Deu-Pons, D. Tamborero, M. P. Schroeder, A. Jene-Sanz, A. Santos, N. Lopez-Bigas, IntOGen-mutations identifies cancer drivers across tumor types, Nature methods 10 (11) (2013) 1081–1082.

[3] S. Razick, G. Magklaras, I. M. Donaldson, iRefIndex: a consolidated protein interaction database with provenance, BMC bioinformatics 9 (1) (2008) 1.

[4] D. Szklarczyk, A. Franceschini, S. Wyder, K. Forslund, D. Heller, J. Huerta-Cepas, M. Simonovic, A. Roth, A. Santos, K. P. Tsafou, et al., STRING v10: protein–protein interaction networks, integrated over the tree of life, Nucleic acids research 43 (D1) (2014) D447–D452.

[5] M. D. Leiserson, F. Vandin, H.-T. Wu, J. R. Dobson, B. R. Raphael, Pan-cancer identification of mutated pathways and protein complexes, Cancer Research 74 (19 Supplement) (2014) 5324–5324.

[6] S. Babaei, M. Hulsman, M. Reinders, J. de Ridder, Detecting recurrent gene mutation in interaction network context using multi-scale graph diffusion, BMC bioinformatics 14 (1) (2013) 29.

[7] A. Cho, J. E. Shim, E. Kim, F. Supek, B. Lehner, I. Lee, MUFFINN: cancer gene discovery via network analysis of somatic mutation data, Genome Biology 17 (1) (2016) 129.

[8] M. Kanehisa, Y. Sato, M. Kawashima, M. Furumichi, M. Tanabe, KEGG as a reference resource for gene and protein annotation, Nucleic acids research (2015) gkv1070.
